# Establishment and prospective validation of an SUV_max_ cutoff value to discriminate clinically significant prostate cancer from benign prostate diseases in patients with suspected prostate cancer by ^68^Ga-PSMA PET/CT: a real-world study

**DOI:** 10.7150/thno.58140

**Published:** 2021-07-25

**Authors:** Jianhua Jiao, Fei Kang, Jingliang Zhang, Zhiyong Quan, Weihong Wen, Xiaohu Zhao, Shuaijun Ma, Peng Wu, Fa Yang, Wei Guo, Xiaojian Yang, Jianlin Yuan, Yongquan Shi, Jing Wang, Weijun Qin

**Affiliations:** 1 Department of Urology, Xijing Hospital, Fourth Military Medical University, Xi'an, Shaanxi, China; 2 Department of Nuclear Medicine, Xijing Hospital, Fourth Military Medical University, Xi'an, Shaanxi, China; 3 Department of Health Services, Health Service Training Base, Fourth Military Medical University, Xi'an 710032, China; 4 Institute of Medical Research, Northwestern Polytechnical University, Xi'an, Shaanxi, China; 5 State Key Laboratory of Cancer Biology, Xijing Hospital, Air Force Military Medical University, Xi'an 710032, China

**Keywords:** SUV_max_, cutoff, prostate cancer, benign prostate hypertrophy, PSMA PET/CT, immunohistochemistry

## Abstract

**Background and Aims:** The aims of this study were to establish a maximum standardized uptake value (SUV_max_) cutoff to discriminate clinically significant prostate cancer (csPCa) from benign prostate disease (BPD) by ^68^Ga-labeled prostate-specific membrane antigen (^68^Ga-PSMA-11) positron emission tomography/computed tomography (PET/CT) in patients with suspected prostate cancer (PCa), and to perform a prospective real-world validation of this cutoff value.

**Methods:** The study included a training cohort to identify an SUV_max_ cutoff value and a prospective real-world cohort to validate it. A retrospective analysis assessed 135 patients with suspected PCa in a large tertiary care hospital in China who underwent ^68^Ga-PSMA-11 PET/CT. All patients were suspected of having PCa based on symptoms, digital rectal examination (DRE), total prostate-specific antigen (tPSA) level, and multiparameter magnetic resonance imaging (mpMRI). The ^68^Ga-PSMA PET/CT results were evaluated using histopathological results from transrectal ultrasound-guided 12-core biopsy with necessary targeted biopsy as references. Patients with Gleason scores (GS) ≥7 from the biopsy results were diagnosed with csPCa, and patients with negative biopsy and follow-up results were diagnosed with BPD. Receiver operating characteristic (ROC) curve analysis was used to identify the optimal SUV_max_ cutoff value. The cutoff value was prospectively validated in 58 patients with suspected PCa. The diagnostic benefits of the cutoff value for clinical decision making were also evaluated.

**Results:** According to ROC curve analysis, the most appropriate SUV_max_ cutoff value for discriminating csPCa from BPD was 5.30 (sensitivity, 85.85%; specificity, 86.21%; area under the curve [AUC], 0.893). The cutoff achieved a sensitivity of 83.33%, a specificity of 81.25%, a positive predictive value (PPV) of 92.11%, a negative predictive value (NPV) of 65.00%, and an accuracy of 82.76% in the prospective validation cohort. Metastases were used as an indicator to reduce false negative results in patients with SUV_max_ ≤ 5.30. In patients without metastases, an SUV_max_ value of 5.30 was also the best cutoff to diagnose localized csPCa (sensitivity, 80.43%; specificity, 86.21%; AUC, 0.852). The cutoff discriminated localized csPCa from BPD with a sensitivity of 76.19%, a specificity of 81.25%, a PPV of 84.21%, an NPV of 72.22%, and an accuracy of 78.38% in the prospective validation cohort. The cutoff, combined with metastases, achieved an accuracy of 89.12% in all patients, increasing accuracy by 8.29% and reducing equivocal results compared with manual reading. There was a strong correlation between SUV_max_ and PSMA expression (*r_s_* = 0.831, *P* < 0.001) and a moderate correlation between SUV_max_ and GS (*r_s_* = 0.509, *P* < 0.001). The PSMA expression and SUV_max_ values of patients with csPCa were significantly higher than those of patients with BPD (*P* < 0.001).

**Conclusion:** We established and prospectively validated the best SUV_max_ cutoff value (5.30) for discriminating csPCa from BPD with high accuracy in patients with suspected PCa. 5.30 is an effective cutoff to discriminate csPCa patients with or without metastases. The cutoff may provide a potential tool for the precise identification of csPCa by ^68^Ga-PSMA PET/CT, ensuring high accuracy and reducing equivocal results.

## Introduction

With nearly 1.4 million new cases in 2020, prostate cancer (PCa) is the second most commonly diagnosed cancer and the fifth leading cause of cancer-associated death in males worldwide 1. Unique from other tumors, prostate tumors require more than a malignant/benign classification. PCa can be divided according to Gleason score (GS) as clinically significant prostate cancer (csPCa, GS = 7-10) and clinically non-significant PCa (cnsPCa, GS = 6). Because the 10- and 15-year actuarial cause-specific survival rates of cnsPCa with active surveillance are as high as 98.1% and 94.3% 2, the recommend treatment strategy for cnsPCa is active surveillance, which is different from the recommended strategy for csPCa 3-5. As a result, the identification of csPCa is a unique clinical need for decision making. ^68^Ga-labeled prostate-specific membrane antigen (^68^Ga-PSMA) positron emission tomography/computed tomography (PET/CT) is a relatively new molecular imaging technique that shows superior performance to conventional imaging techniques in diagnosing and staging PCa, providing us with an opportunity to discriminate patients with csPCa (GS ≥ 7) from those with benign prostate diseases (BPD) 6, 7. Compared with conventional CT and bone scanning,^ 68^Ga-PSMA PET/CT has higher sensitivity and specificity 8. Therefore, ^68^Ga-PSMA PET/CT may accurately discriminate csPCa from BPD, which is currently difficult as patients with BPD exhibit similar results from traditional examinations as patients with csPCa. ^68^Ga-PSMA PET/CT could also provide more information for the primary staging of patients with PCa.

Currently, the evaluation of ^68^Ga-PSMA PET/CT results is still highly dependent on the experience of nuclear medicine experts, and quantitative standards for its parameters are still lacking. Consensus statements on PSMA PET/CT have demonstrated that a cutoff value for the maximum standardized uptake value (SUV_max_) is urgently needed 9. SUV_max_ is the highest signal within a volume of interest (VOI), which has high reproducibility between investigators as it is not dependent on the size of the selected VOI 10. SUV_max_ is appropriate for diagnosing primary PCa because it correlates significantly with PSMA expression 11. For primary PCa, patients with higher GS tend to have stronger PSMA expression 12. Consistent with PSMA expression by immunohistochemistry (IHC), the SUV_max_ of ^68^Ga-PSMA PET/CT is also closely correlated with GS 13-16. In a segment analysis of patients with PCa, ^68^Ga-PSMA PET/CT showed a high detection rate, and the SUV_max_ values of the segments with GS = 7-10 were significantly higher than those of their nearby normal prostate (PN) tissues 13. In a patient analysis, there was a moderate positive correlation between SUV_max_ values and GS, and patients with higher GS had higher SUV_max_ values 14, 15.

However, the previous studies focused on either lesion-based studies to discriminate PCa from BPD or patient-based studies to discriminate patients with high-risk PCa within a PCa cohort 11, 13-17. A large cohort patient-based analysis for the differential diagnosis of csPCa from BPD in patients with suspected PCa by SUV_max_ of ^68^Ga-PSMA PET/CT is essential. A threshold value for the SUV_max_ cutoff is urgently needed to discriminate patients with csPCa from those with BPD in clinical practice.

An appropriate SUV_max_ cutoff value to discriminate patients with csPCa from patients with BPD has not been described. A large cohort study with prospective real-world validation and histopathological results is still lacking. Therefore, the aim of this study was to select and prospectively validate an SUV_max_ cutoff value for discriminating patients with csPCa from those with BPD using pathological results as references. The additional diagnostic benefits of the established SUV_max_ cutoff value for clinical decision making were also evaluated.

## Methods

### Study population

A database from a large tertiary care hospital in China was retrospectively analyzed. All patients with suspected PCa based on symptoms and elevated prostate-specific antigen (PSA) underwent ^68^Ga-PSMA PET/CT from April 2017 to December 2019. Patients were included if they had suspected PCa based on the following examinations: 1) symptoms, 2) digital rectal examination (DRE), 3) total PSA (tPSA) levels, and 4) multiparameter magnetic resonance imaging (mpMRI). Specimens from biopsy were also needed for analysis. Patients with suspicious findings by mpMRI, as well as those with ongoing clinical concern despite a normal mpMRI, underwent ^68^Ga-PSMA PET/CT and biopsy. The exclusion criteria were as follows: 1) treatment, such as androgen deprivation therapy (ADT), radical prostatectomy (RP), radiotherapy, or chemotherapy, was received before ^68^Ga-PSMA PET/CT; 2) the interval between ^68^Ga-PSMA PET/CT and biopsy was longer than 30 days; or 3) GS = 6 (3 + 3). The patients in the prospective validation group were enrolled from the same center from January 2020 to October 2020. The study was approved by the Ethics Committee of Xijing Hospital (approval no. KY20162088-1), and all participating patients provided written informed consent. The research was conducted in adherence with the Declaration of Helsinki and national regulations.

### Study design

Relevant clinical data were collected for patient charts, such as tPSA levels at scan time and GS. All tissues from biopsies were analyzed for PSMA expression by IHC. We recorded the SUV_max_ values and zonal anatomy of primary tumors in ^68^Ga-PSMA PET/CT-positive cases. Metastases, such as lymph node metastases (LNMs), bone metastases (BMs), and visceral metastases, were also recorded for analysis. Patients with cnsPCa (GS = 6) were excluded in the identification of the SUV_max_ cutoff value because they may receive active surveillance to treat indolent tumors 4, 5. However, they were included in the analysis of the correlation between PSMA expression and GS.

### Collection and evaluation of ^68^Ga-PSMA PET/CT images

Patients underwent ^68^Ga-PSMA PET imaging with a Biograph 40 system (Siemens Medical Solutions, Erlangen, Germany). The mean injection activity of ^68^Ga-PSMA PET was 139.72 ± 25.00 MBq. Details are given in the supplementary information. All ^68^Ga-PSMA PET/CT images were evaluated at a single center (Xijing Hospital, Fourth Military Medical University, Xi'an, Shaanxi, China). The ^68^Ga-PSMA PET/CT scans were evaluated by two board-certified nuclear medicine experts (Z.Q. and F.K.) with more than 10 years of experience in reading PET images and one board-certified radiation oncologist (J.W.). The scans were evaluated using a Siemens MIWP workstation (Syngo MIWP; Siemens Medical Solutions, Erlangen, Germany) according to the Joint European Association of Nuclear Medicine and Society of Nuclear Medicine and Molecular Imaging procedure guidelines (version 1.0) 18, 19. The zonal anatomy analysis was based on a method from a previous study 20.

### Histological examination

A transrectal ultrasound-guided 12-core biopsy with necessary additional target biopsy was performed for each patient. All tissues from biopsies were routinely fixed in formalin and processed for hematoxylin-eosin (HE) staining and IHC staining for PSMA. The GS (International Society of Urological Pathology grade) was considered to be the highest score on the biopsy specimens for each patient. As references, the histopathological results were stratified in accordance with the 7^th^ edition of the American Joint Committee on Cancer staging system for PCa 21. The pathological sections were scored according to the consensus of two board-certified genitourinary pathologists, as previously described 22. The specialists were blinded to both the clinical evaluation of the samples from the surgeons and the ^68^Ga-PSMA PET/CT results.

### IHC staining and evaluation

All tissues were fixed in formalin, embedded in paraffin, and routinely processed for IHC staining to evaluate PSMA expression with an anti-PSMA monoclonal antibody (clone 1D6, 1:100, MAB-0575, MXB Biotechnologies), as we previously described 23. PSMA expression was assessed in a semiquantitative manner according to the modified H-score method, adapted from previous studies 24, 25. The staining intensity categories (0 = negative, 1 = weak, 2 = moderate, 3 = strong, and 4 = extremely strong) are shown in Figure S1. A modified H-score ([% weak staining × 1] + [% moderate staining × 2] + [% strong staining × 3] + [% extremely strong staining × 4]) was given by the consensus of two experienced pathologists to determine the overall percentage of PSMA positivity across the entire stained specimen, yielding a score range from 0 to 400 26. Details are given in the supplementary information.

### Intraclass correlation coefficient analysis

The SUV_max_ was measured by two nuclear medicine specialists (Z.Q. and F.K.) with more than ten years of experience in reading PET images. Intraclass correlation coefficient (ICC) analysis of the SUV_max_ values was used to evaluate interobserver reproducibility, as previously reported 27. ICC results were determined to indicate very good agreement if greater than 0.80 27. Details are given in the supplementary information.

### Follow-up

The patients were followed up every 6 months by imaging, biochemistry, and histopathology, consistent with guidelines and previous studies 8, 28. In the follow-up, mpMRI, tPSA measurement, and necessary re-biopsy were performed. All patients were followed up at least once in this study.

### Statistical analysis

Descriptive statistics were calculated and presented as the frequency (percentage) for categorical variables, the mean (standard deviation) for continuous variables with a normal distribution, and the median (quartile) for continuous variables with a skewed distribution. Two-sample *t*-tests were used to assess continuous variables with a normal distribution, and the Wilcoxon signed-rank test was used to assess continuous variables with a skewed distribution. The Mann-Whitney U test was used to compare means from two samples. The correlation between two samples was analyzed by Spearman's ρ test. The receiver operating characteristic (ROC) curve was used to determine the cutoff value of SUV_max_ for diagnosing patients with csPCa. The ICC analysis was performed by reliability analysis. All statistical analyses were conducted using IBM SPSS statistics software, version 23.0 (IBM, Inc., Chicago, IL, USA) and GraphPad Prism software, version 8.0 (GraphPad Software, Inc., La Jolla, CA, USA). All hypothesis tests were two-sided, and *P* < 0.05 was considered to indicate statistical significance.

## Results

### Patient characteristics

**Figure 1:**
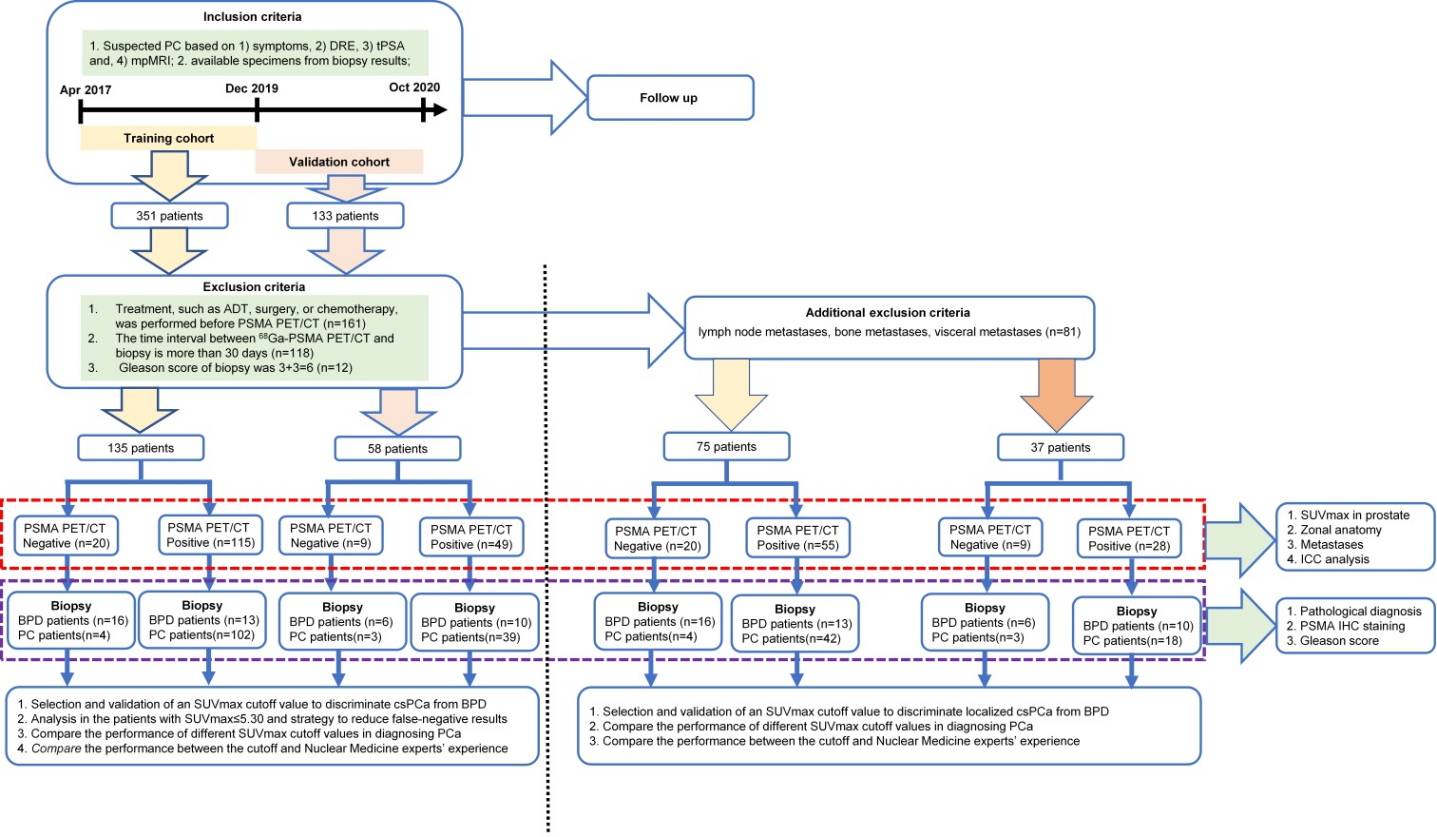
Study design.

According to the inclusion and exclusion criteria, 135 patients were included in the training cohort and 58 patients were recruited for the prospective validation cohort. In the training cohort of 135 patients with suspected PCa, 106 patients were confirmed to have csPCa and the other 29 patients were pathologically diagnosed with BPD (Figure 1). The validation cohort of 58 patients with suspected PCa included 42 with csPCa and 16 with BPD (Figure 1). The patient characteristics are shown in Table 1. The detailed pathological diagnosis of patients with BPD is shown in Table S1. After excluding the patients with metastatic csPCa (mcsPCa; LNMs, BMs, or visceral metastases), the characteristics of the remaining patients are shown in Table S2. As shown Table S3, no significant differences were observed between the training and validation cohorts.

### SUV_max_ is closely correlated with PSMA expression

**Figure 2:**
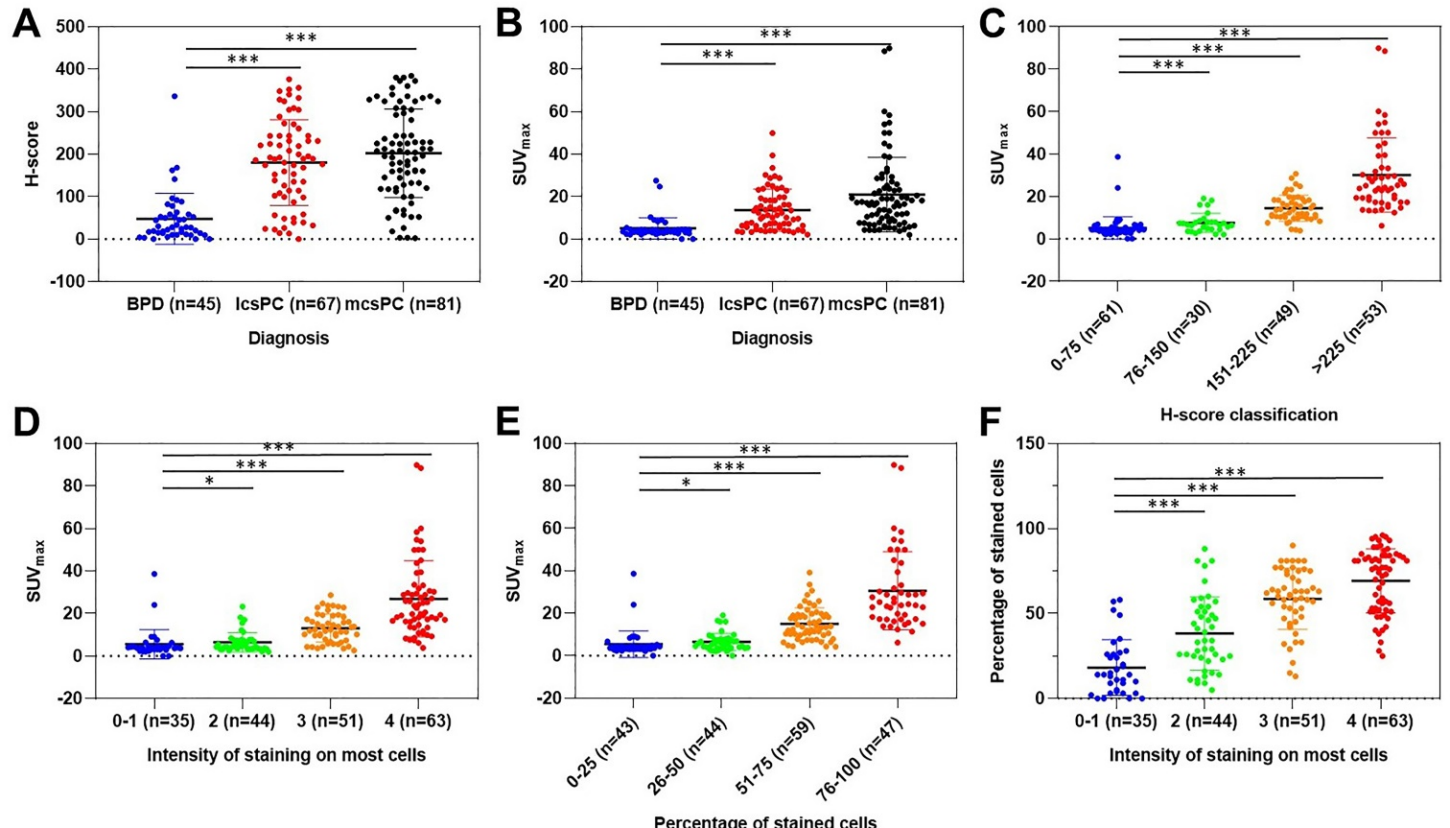
** Scatter dot plots depicting H-score according to pathological diagnosis (A); SUV_max_ according to pathological diagnosis (B), H-score (C), intensity of PSMA staining (D), and percentage of PSMA-stained cells (E); and the percentage of stained cells according to intensity of staining (F).** The vertical borders of the box represent the standard deviation, and the middle bar represents the mean value. The H-scores and SUV_max_ values of patients with lcsPCa and mcsPCa were significantly higher than those with BPD. The SUV_max_ values were higher in prostatic tissues with higher H-scores, intensities of PSMA staining, and percentages of stained cells than in tissues with lower values. The percentage of stained cells was higher in prostatic tissues with more intense PSMA staining. The detailed comparison data are shown in Tables S4A-C (*, *P* < 0.05; **, *P* < 0.01; ***, *P* < 0.001). SUV_max_ was closely positively correlated with PSMA expression, as validated by IHC staining (n = 193).

**Figure 3:**
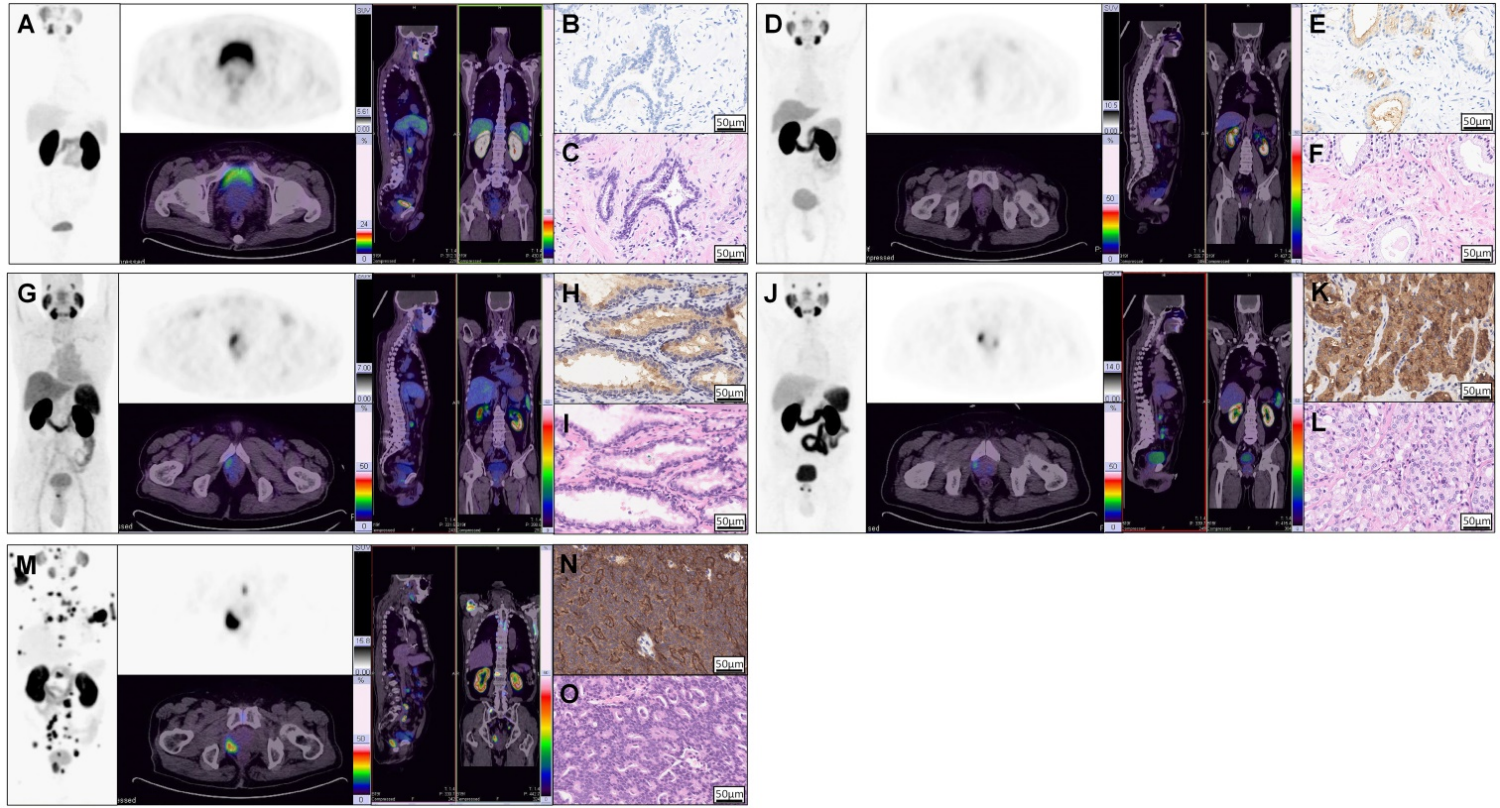
** Example ^68^Ga-PSMA PET/CT images and corresponding IHC and HE staining results showing that SUV_max_ is significantly correlated with PSMA expression in prostatic tissues.** ^68^Ga-PSMA PET/CT images (A, D, G, J, M), PSMA staining results (B, E, H, K, N), and HE staining results (C, F, I, L, O) of one patient with BPD (A-C) and four patients with PCa (D-O). The first patient (A, SUV_max_ = 2.90) was pathologically diagnosed with BPD (B, negative staining, 0% stained cells, tPSA = 19.09 ng/mL), while the remaining four patients (C, E, G, I; SUV_max_ = 3.00, 6.50, 14.50, 60.00, respectively) had pathologically proven PCa (D, F, H, J; GS = 6 (3 + 3), 6 (3 + 3), 7 (4 + 3), 8 (4 + 4); tPSA = 7.43, 19.09, 8.04, 936.10 ng/mL; percentage of stained cells = 15%, 35%, 57%, 95%; intensity of staining = 1, 2, 3, 4). The above representative patients were used to show the close correlation between PSMA expression and SUV_max_. Detailed analysis of all patients is shown in Figure 2.

As we know, PSMA expression on csPCa tissue is significantly higher than that on BPD tissue. To investigate the correlation between SUV_max_ and PSMA expression by IHC staining and to justify SUV_max_ in diagnosing patients with csPCa, we analyzed the distribution of SUV_max_ values according to PSMA expression. As shown in Figure 2A, the H-scores in primary tumors of mcsPCa (205.83 ± 103.43) were higher than those in localized csPCa (lcsPCa, 177.10 ± 98.56), followed by BPD (Table S4A, 45.51 ± 60.17, *P* < 0.001). Consistent with the H-score results, the SUV_max_ values in patients with mcsPCa were also higher than those in patients with BPD (Figure 2B,* P* < 0.001). The SUV_max_ values were positively correlated with H-score, PSMA staining intensity, and percentage of stained cells (Figure 2C-E, Table S4B). A high percentage of stained cells was observed in the group with high staining intensity (Figure 2F, Table S4C). The detailed data of the comparison in Figure 2 are shown in Table S4A-C. To show the close correlation between SUV_max_ and PSMA expression, the ^68^Ga-PSMA PET/CT and IHC results of five representative patients are presented in Figure 3. For all patients, including patients with csPCa or BPD, the SUV_max_ value in the primary tumor was significantly higher in patients with high H-scores and strong PSMA staining intensity and percentage of stained cells than in patients with low values (Table 2). Furthermore, Spearman's ρ test results suggested a strong correlation between SUV_max_ and H-score (Table 2, *r_s_* = 0.831, *P* < 0.001). False negative and false positive results of ^68^Ga-PSMA PET imaging were also observed (Figure S2). Thus, the SUV_max_ value of ^68^Ga-PSMA PET/CT was significantly positively correlated with PSMA expression, as validated by IHC staining. Next, in order to further prove the potential of SUV_max_ to diagnose patients with csPCa, we analyzed the correlation between SUV_max_ and GS to investigate the potential of an SUV_max_ cutoff to discriminate csPCa from BPD.

### SUV_max_ is closely correlated with GS

**Figure 4:**
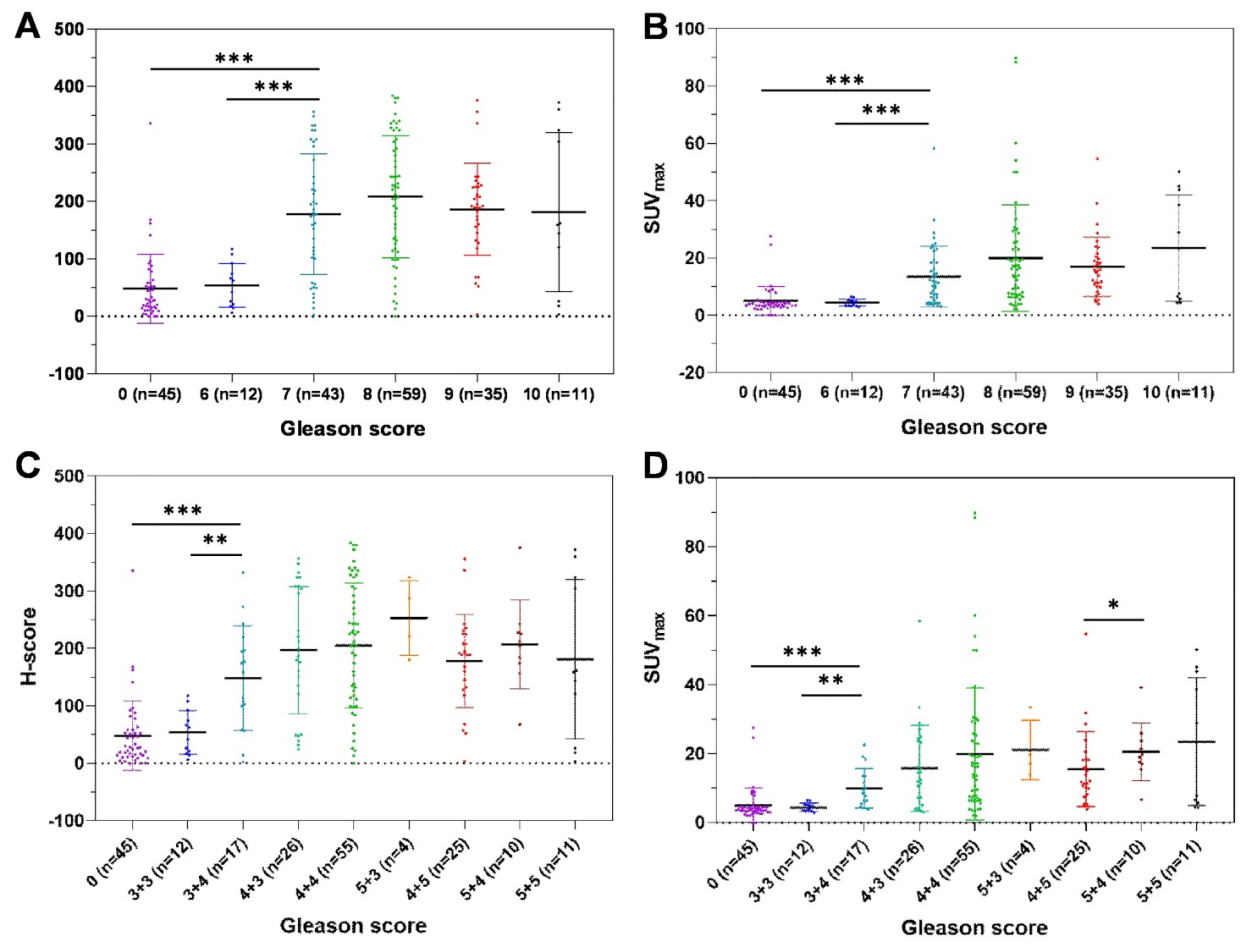
** Correlations between GS and H-score (A, C) and SUV_max_ (B, D).** The H-scores and SUV_max_ values of patients with GS = 7 PCa were significantly higher than those with GS = 6 PCa or BPD (GS=0). (*, *P* < 0.05; **, *P* < 0.01; ***, *P* < 0.001).

To investigate the correlation between SUV_max_ and GS, we analyzed the distribution of SUV_max_, validated by H-score, according to GS. Spearman's ρ test results suggested a moderate correlation between SUV_max_ and GS (Table 3, *r_s_* = 0.509, *P* < 0.001). The H-scores and SUV_max_ values of patients with GS = 7 were significantly higher than those of patients with BPD and patients with GS = 6 PCa (Figure 4A-B, *P* < 0.001). In a detailed GS analysis, the H-scores and SUV_max_ values of patients with GS = 7 (3 + 4) were higher than those of patients with BPD and patients with GS = 6 PCa (Figure 4C-D, Table S5A-B, *P* < 0.001). Aside from the difference in SUV_max_ values between GS = 4 + 5 and GS = 5 + 4, no significant difference was found in other adjacent GS groups. In conclusion, the SUV_max_ value and H-score of primary tumors are closely correlated with GS. It is very feasible to generate a cutoff value for SUV_max_, validated by H-score, to discriminate patients with csPCa from those with BPD because the SUV_max_ values of patients with csPCa (GS ≥ 7) were significantly higher than those of patients with BPD. Next, we identified and validated an SUV_max_ cutoff value to discriminate csPCa from BPD.

### An SUV_max_ cutoff value is established and validated to discriminate csPCa from BPD

**Figure 5:**
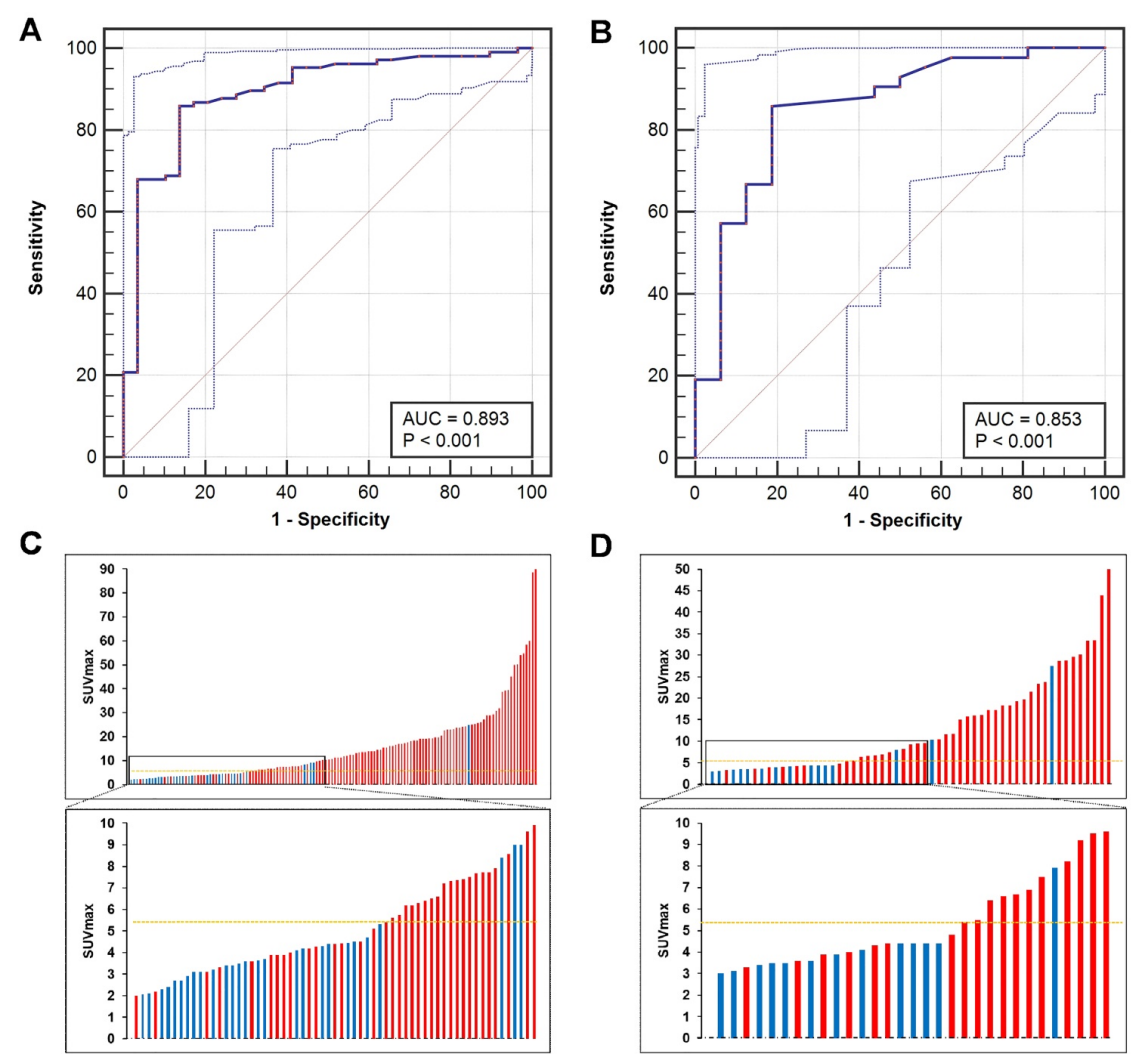
** ROC curves for diagnosing patients with csPCa.** (A, C) The SUV_max_ cutoff value of 5.30 yielded a sensitivity of 85.85% and a specificity of 86.21% in the training cohort (AUC = 0.893, Youden's index = 0.721). (B, D) The cutoff of 5.30 achieved a sensitivity of 83.33% and a specificity of 81.25% in the prospective validation cohort. The top and bottom ROC curves represent the upper and lower bounds of the 95% confidence interval of the middle bound, respectively.

ROC curve analysis was used to identify the best SUV_max_ cutoff value to discriminate patients with csPCa from those with BPD using pathological results as references. In the training cohort, the ROC curve analysis showed that the best SUV_max_ cutoff value to discriminate csPCa from BPD was 5.30, and this value had a sensitivity of 85.85%, a specificity of 86.21%, a positive predictive value (PPV) of 95.79%, a negative predictive value (NPV) of 62.50% and an accuracy of 85.93% (Figure 5A, C, AUC = 0.893,* P* < 0.001). In the prospective validation cohort, the cutoff value of 5.30 achieved a sensitivity of 83.33%, a specificity of 81.25%, a PPV of 92.11%, an NPV of 65.00%, and an accuracy of 82.97% (Figure 5B, D, AUC = 0.853). Comparisons between 5.30 and other reported cutoff values are shown in Table 3 6, 11, 14, 29-31. The cutoff value of 5.30 achieved the highest accuracy in all patients of the training and validation cohorts to discriminate csPCa from BPD.

### The patients with SUV_max_ ≤ 5.30 is analyzed to reduce the false negative rate

**Figure 6:**
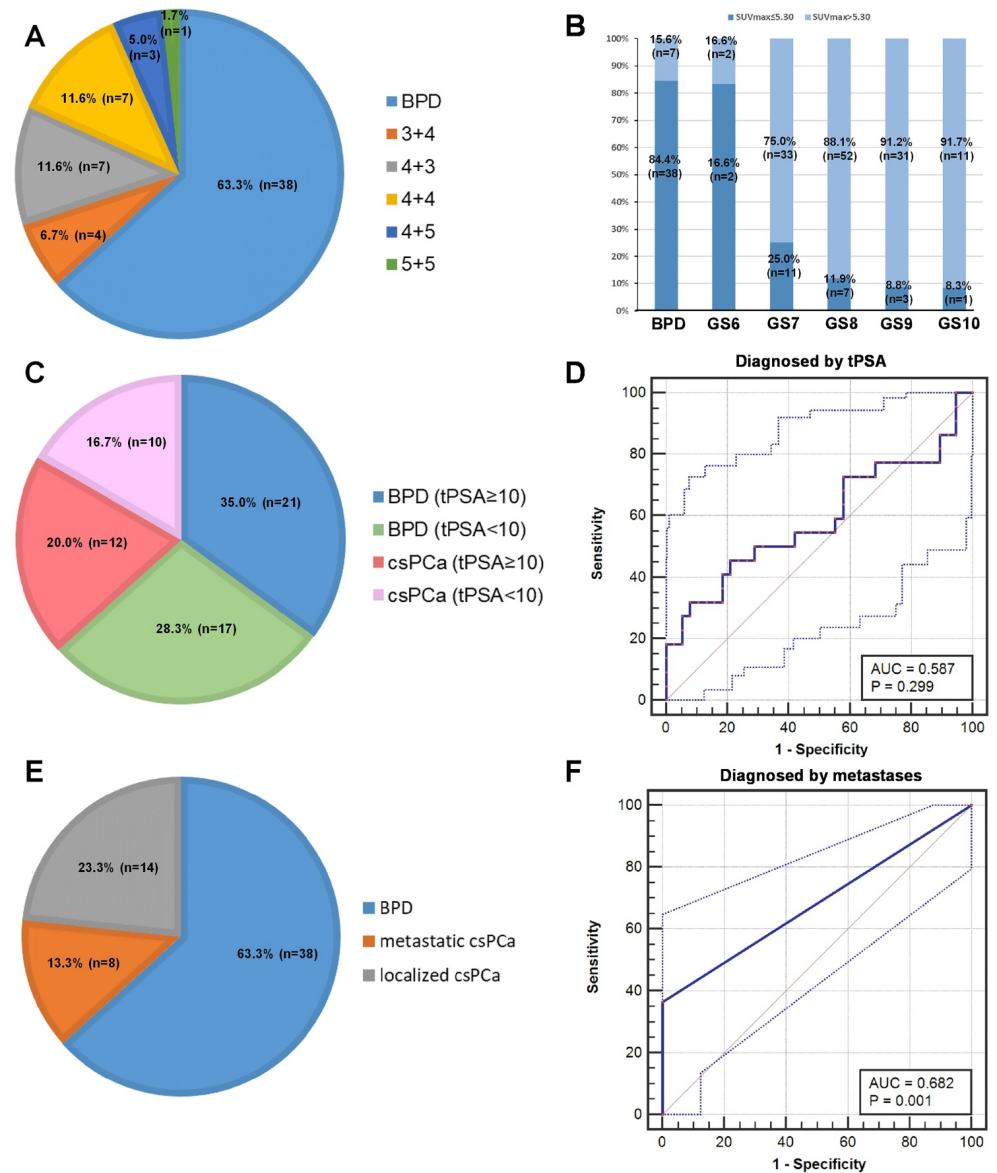
** Analysis of patients with SUV_max_** ≤ **5.30.** (A) Pathological diagnoses of patients with SUV_max_ ≤ 5.30. (B) Percentage of patients with SUV_max_ ≤ 5.30 in each GS group. A higher percentage of patients with low GS had an SUV_max_ ≤ 5.30 than patients with high GS. (C) tPSA levels of patients with SUV_max_ ≤ 5.30. (D) ROC curves for diagnosing csPCa by tPSA. (E) Metastatic status of patients with SUV_max_ ≤ 5.30. (F) ROC curves for diagnosing patients with csPCa by metastatic status. The top and bottom ROC curves represent the upper and lower bounds of the 95% confidence interval of the middle bound, respectively.

To investigate strategies to reduce the false negative rate, we further analyzed the patients with SUV_max_ ≤ 5.30 in detail. In the patients with SUV_max_ ≤ 5.30, 63.33% (38/60) of the patients were diagnosed pathologically with BPD, and 36.67% (22/60) of the patients were diagnosed with csPCa (Figure 6A). As shown in Figure 6B, patients with PCa and lower GS more frequently had SUV_max_ ≤ 5.30, i.e., a higher false negative rate, than patients with higher GS. We tried to use tPSA levels and metastases to further diagnose patients with csPCa and reduce false negative results. In 60 patients with false negative diagnoses, 54.55% (12/22) of patients with csPCa had tPSA ≥10 ng/mL, and 55.26% (21/38) of patients with BPD had tPSA ≥10 ng/mL. The ROC curve showed that tPSA level could not be used to discriminate patients with csPCa from those with BPD (Figure 6C-D, AUC = 0.587,* P* = 0.299). This is partly due to the moderate correlation between SUV_max_ and tPSA (Table 3, *r_s_* = 0.445, *P <* 0.001). For the metastases analysis, 8 patients with csPCa showed metastases, including LNMs, BMs, and visceral metastases, by ^68^Ga-PSMA PET/CT. The ROC curve showed that metastases can be used to decrease false negative results in patients with SUV_max_ ≤ 5.30 (Figure 6E-F, AUC = 0.682,* P* =0.001). Using metastases as references, 38.10% (8/21) of false negative results could be avoided (Figure 6E-F). Taking an SUV_max_ cutoff value of 5.30 and metastases into full consideration, the diagnostic accuracy achieved was 89.12% (172/193) in diagnosing patients with csPCa by ^68^Ga-PSMA PET/CT (Table 3). Thus, metastases can be used to reduce the false negative rate of using the cutoff alone for diagnosis. Next, we further investigated whether an SUV_max_ cutoff value can be identified to diagnose patients with lcsPCa.

### An SUV_max_ cutoff value is established and validated to discriminate lcsPCa from BPD

**Figure 7:**
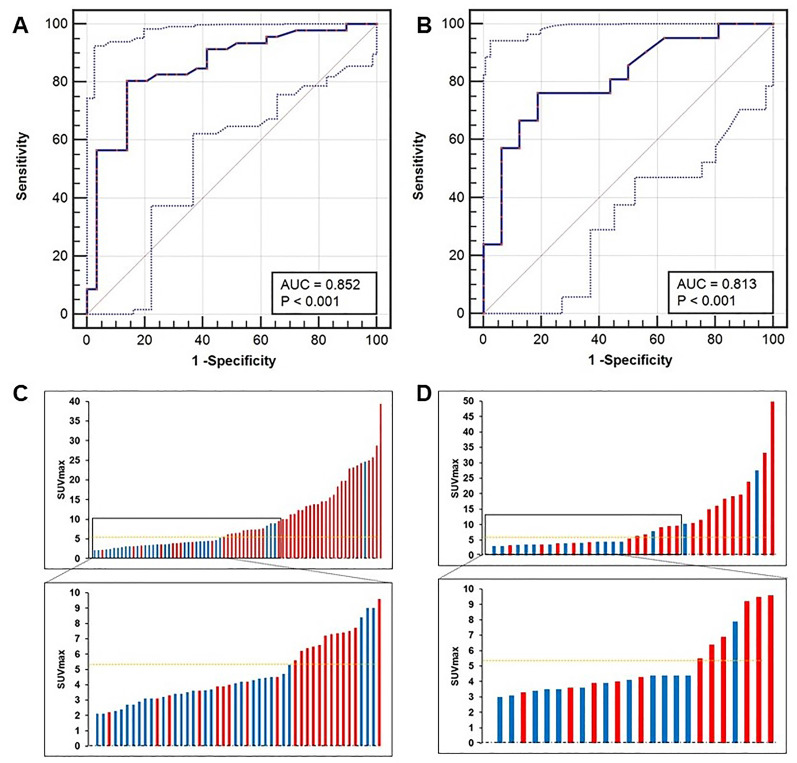
** ROC curves of patients with lcsPCa.** (A, C) The SUV_max_ cutoff value of 5.30 yielded a sensitivity of 80.43% and a specificity of 86.21% in the training cohort (AUC = 0.852, Youden's index = 0.666). (B, D) The cutoff of 5.30 achieved a sensitivity of 76.19% and a specificity of 81.25% in the prospective validation cohort. The top and bottom ROC curves represent the upper and lower bounds of the 95% confidence interval of the middle bound, respectively.

We next identified the best SUV_max_ cutoff value to diagnose patients with lcsPCa. By ROC curve analysis, the best SUV_max_ cutoff value to discriminate lcsPCa from BPD was also 5.30, and this value had a sensitivity of 80.43%, a specificity of 86.21%, a PPV of 90.24%, an NPV of 73.53%, and an accuracy of 82.67% (Figure 7A, C, AUC = 0.852, *P* < 0.001). In the prospective validation group, the cutoff value of 5.30 achieved a sensitivity of 76.19%, a specificity of 81.25%, a PPV of 84.21%, an NPV of 72.22%, and an accuracy of 78.38% (Figure 7B, D). Comparisons of the diagnostic efficiency of 5.30 with other reported cutoff values are shown in Table S6 6, 11, 14, 29-31. The SUV_max_ cutoff value of 5.30 achieved the highest accuracy for discriminating lcsPCa in both the training cohort and the validation cohort. Thus, consistent with the best cutoff value for diagnosing all patients with csPCa, 5.30 is also the most appropriate SUV_max_ cutoff value for discriminating patients with lcsPCa from those with BPD by ^68^Ga-PSMA PET/CT. Next, we investigated whether this cutoff can benefit clinical decision making.

### The SUV_max_ cutoff may benefit clinical diagnosis by ^68^Ga-PSMA PET/CT

**Figure 8:**
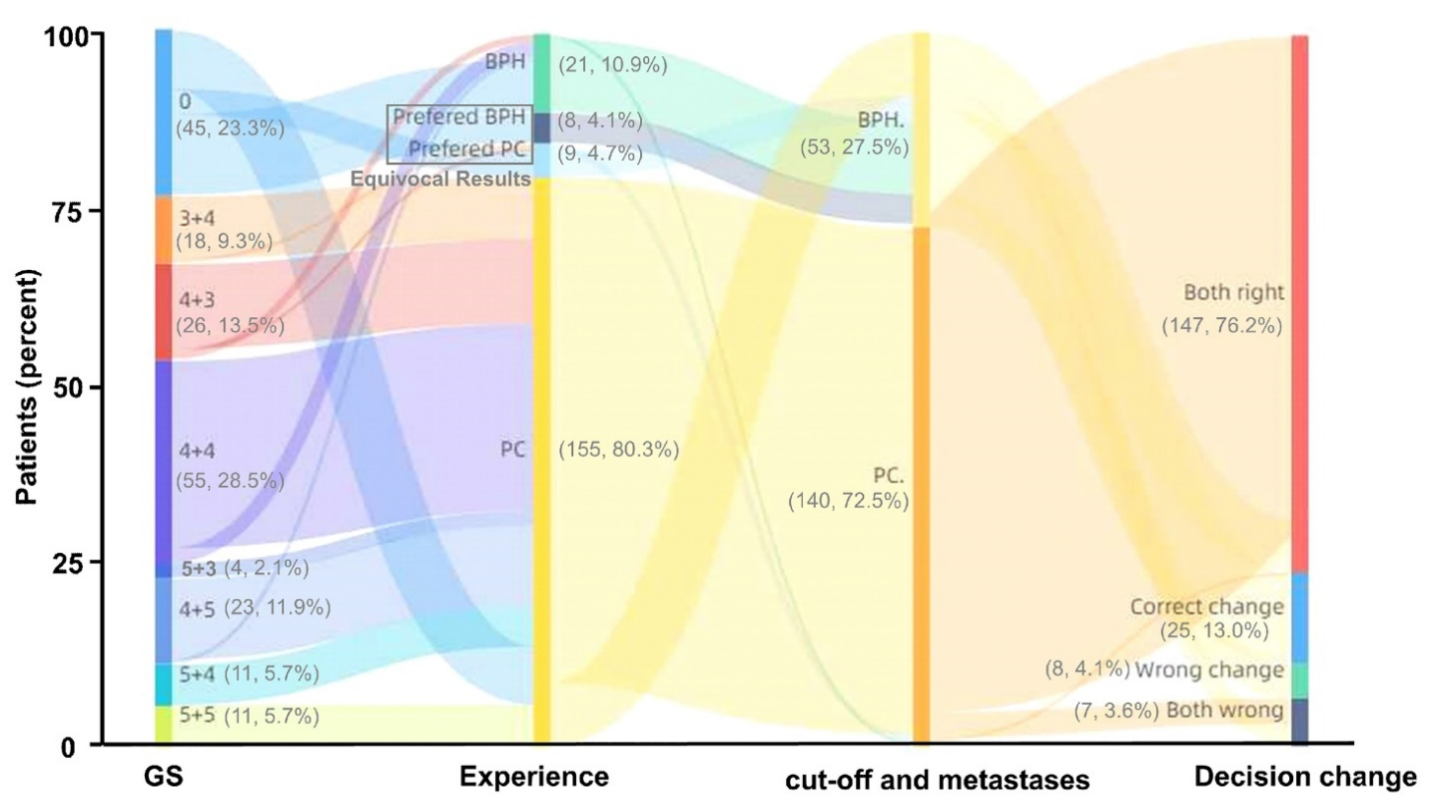
** Changes in clinical diagnosis based on ^68^Ga-PSMA PET/CT between nuclear medicine expert experience and the combination of SUV_max_ cutoff and metastases.** Compared with nuclear medicine expert experience, using the cutoff and metastases improved the diagnostic accuracy of clinical decision making with ^68^Ga-PSMA PET/CT by 8.81% (17/193).

Diagnosis by ^68^Ga-PSMA PET/CT is highly dependent on the experience of nuclear medicine experts. Therefore, we next investigated whether the identified SUV_max_ cutoff value has the potential to benefit this diagnostic procedure. In all 193 patients, the diagnostic accuracy of the nuclear medicine expert decision was 80.83% (156/193), and 8.81% (17/193) of the results were relatively equivocal. Using the SUV_max_ cutoff value of 5.30 and metastases as references, the diagnostic accuracy reached 89.12% (172/193), and 8.29% (16/193) more patients in this group were diagnosed correctly by ^68^Ga-PSMA PET/CT compared with manual reading (Figure 8). Thus, the SUV_max_ cutoff can benefit clinical decision making by ^68^Ga-PSMA PET/CT, greatly reducing equivocal results and improving the accuracy of clinical diagnosis.

### Follow-up, ICC analysis, and zonal anatomy analysis

To avoid false negative biopsy results and interobserver differences in measuring SUV_max_, follow-up and ICC analysis were also performed. Zonal anatomy analysis was also used to evaluate cutoffs in different segments of the prostate. In the follow-up of at least six months, no biopsy-negative BPD patients were confirmed to have PCa. In 48 patients who received RP, the GS in 81.25% (39/48) of the patients was unchanged, while 14.58% (7/48) of patients had upgraded GS and 4.17% (2/48) of patients had downgraded GS (Table S7). In the ICC analysis, two nuclear medicine experts achieved very good agreement on the measurement of SUV_max_ (ICC = 0.993, *P* < 0.001, Table S8). After considering the morphology and location of ^68^Ga-PSMA uptake, the patients were categorized as PSMA PET negative, csPCa with ^68^Ga-PSMA uptake in the peripheral segments alone, and csPCa with ^68^Ga-PSMA uptake in the central segments. The best SUV_max_ cutoff value to diagnose peripheral csPCa was 4.70, and the cutoff to recognize csPCa involved in the central segments was 9.0 (Figure S3). Follow-up showed that the biopsy GS was accurate enough for this study, and the ICC analysis showed excellent agreement between different observers in measuring SUV_max_.

## Discussion

This study represents the largest clinical study to identify and prospectively validate an SUV_max_ cutoff value for discriminating patients with csPCa from those with BPD, using pathological results as references. In this study, we identified and prospectively validated an SUV_max_ cutoff value to discriminate patients with csPCa from those with BPD in patients with suspected PCa based on conventional examinations. Compared with clinical diagnosis based on the experience of nuclear medicine experts, the diagnostic accuracy increased from 80.83% (156/193) to 89.12% (172/193) and relatively equivocal results (8.81%) were reduced.

PSMA, a type II membrane protein with folate hydrolase activity, is expressed at a significantly higher level in more than 90.00% of PCa tissues than BPD tissues 12, 32-34. The SUV_max_ of ^68^Ga-PSMA is closely related to the expression of PSMA 11, 22. The SUV_max_ value is higher in PCa tissues than in BPD tissues because of the higher expression of PSMA, as validated by IHC staining 11. The mean SUV_max_ value of PCa tissues (14.10 ± 15.60) has been found to be significantly higher than that of PN tissues (2.40 ± 0.60, *P* < 0.001) 11. In another study, the average SUV_max_ value was 11.00 ± 7.80 in PCa tissues and 2.70 ± 0.90 in PN tissues (*P* < 0.001) 29. However, due to individual differences, low to moderate PSMA expression is also observed in BPD tissues 33, and these tissues may even have high PSMA expression 12. Hence, relatively low SUV_max_ values in BPD tissues can also be measured by ^68^Ga-PSMA PET/CT. The SUV_max_ values of benign prostatic hyperplasia (BPH) tissues range from 3.20 to 5.80, and the SUV_max_ values of PN tissues range from 2.50 to 6.60 15, 35. Other studies demonstrated that the median SUV_max_ values in PN tissues range from 2.4 to 5.5, with a maximum value of 8.3 10, 36, 37. The subjects of the above studies were patients with PCa, but the differential diagnosis of BPD is also important. In our study, compared with patients with BPD, patients with csPCa had higher SUV_max_ values in their primary lesions, as measured by ^68^Ga-PSMA PET/CT and validated by IHC staining (Figure 2), which is in accordance with the results of previous studies 38, 39. In summary, csPCa tissues are known to have higher SUV_max_ values than BPD tissues, but a cutoff to discriminate patients with csPCa from those with BPD was still unidentified.

In ^68^Ga-PSMA PET/CT imaging, an appropriate SUV_max_ cutoff value is important for the differential diagnosis of patients with csPCa from those with BPD 40. In our study, the validated SUV_max_ cutoff value of 5.30 enabled the diagnosis of patients with csPCa with high sensitivity and specificity. Several previous studies generated SUV_max_ cutoff values to discriminate PCa tissues from their nearby PN tissues, but no cutoff value to discriminate patients with csPCa from those with BPD was previously identified. The first previous study identified an SUV_max_ value of 3.20 that demonstrated a high sensitivity of 94.3% and a high specificity of 100%, without histopathological results and IHC validation 29. The same research group also reported the first pathologically validated cutoff of 3.15 for discriminating PCa tissues from nearby PN tissues, and this cutoff had a sensitivity of 97.0% and a specificity of 90.0% in 31 patients with PCa 11. Another study indicated that an SUV_max_ cutoff value of 4.00 achieved a sensitivity of 88.0%, a specificity of 86.5%, and an accuracy of 87.5% 13. However, this cutoff was identified from 132 segments in 6 patients with high-risk PCa 13. Ferraro et al. also assessed PSMA-expressing tumors using a background-based threshold set to an SUV_max_ cutoff value of 4.00 31. Donato et al. identified an SUV_max_ cutoff value of 6.30 for the detection of csPCa lesions with 100.0% specificity and 60.1% sensitivity (AUC = 0.788) 17. Furthermore, Fendler et al. identified an optimal SUV_max_ cutoff value of 6.50 for discriminating histopathologically positive and negative segments (AUC = 0.84, *P* < 0.001), which yielded a sensitivity of 67% and a specificity of 92% 30. In discriminating patients with csPCa (GS ≥ 7) from patients with cnsPCa (GS = 6), an SUV_max_ cutoff of 3.95 achieved 94% sensitivity and 100% specificity 41. The optimal SUV_max_ cutoff of 6.70 achieved a sensitivity of 88% and a specificity of 96% in discriminating patients with csPCa from those with cnsPCa by PSMA PET/MRI 6. In comparison with the cutoffs generated in the above studies, 5.30 is still the best cutoff for discriminating patients with BPD from those with or without metastases, which is similar to a previous study conducted by Liu et al. 42 (Table 3, Table S6). We found that an SUV_max_ cutoff value could be identified to discriminate patients with csPCa (GS ≥ 7) from those with BPD because of their significant differences in PSMA expression and SUV_max_ values.

As shown in Figure 4, the differences in SUV_max_ values between different GS groups showed that it is feasible to select a cutoff to discriminate patients with csPCa from those with BPD, but it would be difficult to generate a cutoff to further discriminate patients with csPCa by GS. Although the SUV_max_ values in patients with GS = 9 (5 + 4) were higher than those in patients with GS = 9 (4 + 5), there were no significant differences between these patients and other GS groups among patients with csPCa (Figure 4D). In a correlation analysis between SUV_max_ and GS, our study showed that the SUV_max_ values in patients with csPCa (GS ≥ 7) were significantly higher than those in patients with BPD or cnsPCa (GS = 6), but no significant differences existed between other GS groups among patients with csPCa. This result is consistent with previous studies. A few previous studies demonstrated that ^68^Ga-PSMA PET could discriminate patients with high-risk and low-risk PCa. In an analysis of patients with PCa, the SUV_max_ values of patients with GS ≥ 7b (4 + 3) were significantly higher than those of patients with GS ≤ 7a (3 + 4) 14, 16. Another study showed that SUV_max_ is significantly higher in tumors with GS > 7 (8, 9, 10) than in primary tumors with GS ≤ 7 (6, 7a, 7b) 15. In this study, the SUV_max_ values of patients with high-risk PCa (GS ≥ 8) were higher than those of patients with low-to-intermediate-risk PCa (GS < 8) (Figure S4A-B). The SUV_max_ cutoff value to diagnose patients with high-risk PCa was 5.30 in all patients (AUC = 0.779, Figure S4C), while the cutoff was 6.50 in all patients with PCa (AUC = 0.685, Figure S4D). The AUCs of these cutoffs were lower than that of the cutoff to discriminate csPCa from BPD because the SUV_max_ difference mainly exists between the GS = 6 and GS = 7 groups (*P* < 0.001), rather than the GS = 7 and GS ≥ 8 groups (*P* = 0.025). In this study, the SUV_max_ of the GS ≥ 8 group was slightly higher than that of the GS = 7 group (*P* = 0.025, Figure S4B). This may further explain why there was no significant difference in ^68^Ga-PSMA PET positivity between patients with GS ≤ 7 and GS ≥ 8 PCa 43. This may partly explain why a higher SUV_max_ value can be used for the prediction of pathological upgrading, especially for patients with a lower tumor grade at mpMRI targeted biopsy 44. In summary, an SUV_max_ cutoff value can be identified to discriminate patients with csPCa from those with BPD because the jump in PSMA expression mainly exists between patients with GS = 7 csPCa and BPD. This may partly explain why the accuracy of cutoffs to diagnose patients with high-risk PCa (GS ≥ 8) were relatively low.

In our study, SUV_max_ > 5.30 was the most appropriate cutoff value to discriminate csPCa from BPD, facilitating a standardized approach for reporting findings from ^68^Ga-PSMA PET/CT imaging, which is urgently needed 45. Furthermore, we validated the performance of this cutoff in a prospective validation cohort. For patients with SUV_max_ ≤ 5.30, patients with a lower GS had a higher false negative rate, but we found that metastases identified by ^68^Ga-PSMA PET/CT could be used to reduce false negative results, which is inconsistent with one previous study 15. In this study, more patients with PCa in the later stages were included than in the previous study. The accuracy of SUV_max_ > 5.30 was 84.97% in all patients, and the accuracy reached 89.12% after incorporation of metastases (Table 3). The SUV_max_ cutoff value for patients with lcsPCa was 5.30, which was consistent with the cutoff value for discriminating all patients with csPCa from those with BPD. This result is because patients with LNMs or BMs tend to have SUV_max_ values far above 5.30. For patients with lcsPCa, SUV_max_ may predict adverse pathological outcomes and progression-free survival 16. From zonal anatomy analysis, the cutoff for peripheral csPCa was 4.70, and that for csPCa involving the central segments was 9.00 (Figure S3). The peripheral and central segments of the prostate were divided by ^68^Ga-PSMA PET/CT, as previously reported 20. The cutoff for csPCa involving the central segments was higher partly because most PCa originates from the peripheral segments and BPH usually occurs in the transitional zone, which is in the central and nearby central segments. Regarding morphology, Gao et al. reported that ^68^Ga-PSMA PET/CT can identify aggressive cribriform morphology in PCa, and an SUV_max_ cutoff value of 10.90 achieved a sensitivity of 76% and a specificity of 86% in a per-patient analysis 46. In addition to SUV_max_, the combination of morphology and location may further improve the diagnostic accuracy of ^68^Ga-PSMA PET/CT. In this study, no obvious significant differences were observed between the training and validation cohorts (Table S3), so we concluded that the cutoff value (5.30) was validated effectively in the validation cohort. In summary, metastases can be used to reduce false negative results from the SUV_max_ cutoff. The cutoff values in different segments of the prostate were different, and whether location and morphology can further increase diagnostic accuracy still needs to be investigated.

Currently, there is a shift from ^68^Ga- to ^18^F-labeled PSMA agents, and a cutoff to diagnose csPCa can also be established for ^18^F-labeled PSMA PET. Although all Glu-urea-based PSMA-targeted tracers share a similar distribution as physiological tracers, their cutoffs may be affected by their different distributions and excretions. The cutoffs for ^68^Ga-PSMA-11 and ^18^F-DCFPyL might be similar because they share a similar physiological uptake, and the aorta can be used as a benchmark to assess lesions based on SUV_max_ 47. The cutoff for ^18^F-PSMA-1007 might be different from those of the above two tracers due to its hepatobiliary clearance 48. The SUV_max_ cutoffs for several other PSMA ligands (e.g., ^68^Ga-PSMA I&T, ^18^F-rh-PSMA-7) available for PET imaging still need further investigation 47. Although SUV_max_ is an important parameter, more parameters may facilitate a more accurate diagnosis. In quantitative PSMA PET analysis, machine learning-based analysis of quantitative ^18^F-DCFPyL PET can predict metastatic disease or high-risk PCa with GS ≥ 8 49. Radiomic features from PSMA PET images indicated that the texture feature quantization algorithm + short zones high gray-level emphasis (QSZHGE) can discriminate GS = 7 and GS ≥ 8 PCa tumors 50. Furthermore, the lesion-to-background ratio of SUV_max_ in ^68^Ga-PSMA PET/MRI may improve clinical applicability compared with absolute SUV_max_ 51. In summary, the cutoffs for different radiotracers still need further investigation due to their different distributions and excretions, and machine learning-based analysis can further help recognize more useful features.

Among BPD, BPH is the most common differential diagnosis. For the patients with BPD in this study, BPH was the most common pathological result, while other benign diseases, including prostatitis (chronic/acute), necrosis, calcification, and interstitial hypertrophy, were also observed (Table S7). One previous study reported that there was no correlation between tumor size and SUV_max_ 11. In our study, the correlation between SUV_max_ and tumor size was insignificant (*P* = 0.061, Figure S5), but PCa tumors with larger diameters tended to have higher SUV_max_ values in the patients with PCa who received RP (Spearman's ρ, *r_s_* = 0.332, *P* < 0.001).

Our study has some limitations. The first limitation is that the data for this study came from a single center. However, the observed relationship between SUV_max_ and GS is consistent with previous studies. We also performed good quality control and ICC analysis of SUV_max_ to ensure the reliability of the study. Another limitation is possible selection bias. ^68^Ga-PSMA PET/CT was not used to screen patients with csPCa but for further accurate diagnosis after conventional examinations. However, ^68^Ga-PSMA PET/CT was used following mpMRI, the standard-of-care according to current guidelines, and the promising results here provide important data for future prospective clinical trials. The identified cutoff can greatly help clinical diagnosis, including increasing diagnostic accuracy and reducing equivocal results, if validated in multicenter studies. Additionally, the zonal anatomy analysis of different cutoffs was roughly based on CT images. A more detailed zonal anatomy analysis may be conducted using PSMA PET/MRI in a future study.

## Conclusions

In conclusion, our study is the largest study with prospective real-world validation assessing the optimal SUV_max_ cutoff value, using pathological results as references, for discriminating patients with csPCa from those with BPD. We established and prospectively validated the best SUV_max_ cutoff value (5.30) for discriminating csPCa from BPD with high accuracy. 5.30 is also an effective cutoff for discriminating csPCa patients with or without metastases. The cutoff may provide a potential tool for the precise identification of csPCa by ^68^Ga-PSMA PET/CT, ensuring high accuracy and reducing equivocal results. If the cutoff value can be validated in a larger multicenter prospective study, it could be applied to diagnose patients with csPCa more accurately and efficiently than conventional examinations.

## Supplementary Material

Supplementary methods, figures and tables.

## Figures and Tables

**Table 1:** Characteristics of patients with BPD or csPCa.

Characteristic	Training cohort (n = 135)					Validation cohort (n = 58)			
	BPD	csPCa	*χ/z*	*P*		BPD	csPCa	*χ/z*	*P*
**n (%)**	29 (21.5)	106 (78.5)	——	——		16 (27.6)	42 (72.4)	——	——
**Mean age, y**	68.21 ± 9.37	70.16 ± 8.28	——	<0.001		64.56 ± 10.83	70.55 ± 9.72	——	0.054
**Mean acquisition time, min after injection**	61.79 ± 9.90	65.83 ± 13.30	——	0.182		65.56 ± 12.64	65.62 ± 13.11	——	0.896
**Mean interval between biopsy and PSMA PET/CT, d**	10.04 ± 6.15	9.51 ± 6.38	——	0.562		10.07 ± 6.64	10.81 ± 7.04	——	0.972
**Mean H-score**	41.44 ± 39.93	200.04 ± 102.37	-6.874	<0.001		59.25 ± 85.17	172.07 ± 101.77	-3.845	<0.001
**Median tPSA, ng/mL (P_25_-P_75_)**	11.73 (7.10-14.90)	32.92 (10.01-175.73)	-3.568	<0.001		9.72 (7.46-13.56)	40.84 (13.66-83.96)	-3.584	<0.001
≤4, n (%)	4/135 (3.0)	18/135 (13.3)	——	——		1/58 (1.7)	2/58 (3.4)	——	——
4-10, n (%)	6/135 (4.4)	9/135 (6.7)	——	——		8/58 (13.8)	5/58 (8.6)	——	——
10-20, n (%)	14/135 (10.4)	17/135 (12.6)	——	——		5/58 (8.6)	9/58 (15.5)	——	——
>20, n (%)	5/135 (3.7)	62/135 (45.9)	——	——		2/58 (3.4)	26/58 (44.8)	——	——
GS, n (%)	——	106 (100.0)	——	——		——	42 (100.0)	——	——
7 (3 + 4)	——	11/106 (10.4)	——	——		——	7/42 (16.7)	——	——
7 (4 + 3)	——	21/106 (19.8)	——	——		——	5/42 (11.9)	——	——
8 (4 + 4)	——	41/106 (38.7)	——	——		——	14/42 (33.3)	——	——
8 (5 + 3)	——	3/106 (2.8)	——	——		——	1/42 (2.4)	——	——
9 (4 + 5)	——	14/106 (13.2)	——	——		——	9/42 (21.4)	——	——
9 (5 + 4)	——	9/106 (8.5)	——	——		——	2/42 (4.8)	——	——
10 (5 + 5)	——	7/106 (6.6)	——	——		——	4/42 (9.5)	——	——
**lcsPCa, n (%)**	——	46/106 (43.4)	——	——		——	21/42 (50.0)	——	——
**mcsPCa, n (%)**	——	60/106 (56.6)	——	——		——	21/42 (50.0)	——	——
Lymph node	——	44/106 (41.5)	——	——		——	13/42 (31.0)	——	——
Bone	——	42/106 (39.6)	——	——		——	17/42 (40.5)	——	——
Visceral	——	9/106 (8.5)	——	——		——	1/42 (2.4)	——	——

* Statistically significant; average age, acquisition time, and interval were compared using independent samples *t*-tests; average H-score and tPSA were compared using Wilcoxon W tests. Mean values are presented as mean ± SD.

**Table 2:** Correlations between SUV_max_ and H-score, tPSA, and GS.

	*r_s_*	*P*
SUV_max_ vs. H-score	0.831	<0.001*
SUV_max_ vs. GS	0.509	<0.001*
SUV_max_ vs. tPSA level	0.445	<0.001*

* Statistically significant; Spearman's ρ test.

**Table 3:** Sensitivity, specificity, PPV, NPV, and accuracy of ^68^Ga-PSMA PET/CT in detecting csPCa.

	Sensitivity (%)	Specificity (%)	PPV (%)	NPV (%)	Accuracy (%)
**All patients (BPD or csPCa, n = 193)**					
Cutoff > 5.30 and metastases	90.54	84.44	95.04	73.08	89.12
Cutoff > 5.30	85.14	84.44	94.74	63.33	84.97
Cutoff > 3.20 11, 29	97.97	31.11	82.39	82.35	82.38
Cutoff > 4.00 14, 31	91.22	55.56	87.10	65.79	82.90
Cutoff > 6.50 30	77.70	84.44	94.26	53.52	79.27
Cutoff > 6.70 6	75.68	84.44	94.12	51.53	77.72
**Training cohort (n = 135)**					
Cutoff > 5.30 and metastases	91.51	86.21	96.04	73.53	90.37
Cutoff > 5.30	85.85	86.21	95.79	62.50	85.93
Cutoff > 3.20 11, 29	97.17	27.93	85.12	78.57	84.44
Cutoff > 4.00 14, 31	91.51	56.82	88.99	65.38	84.44
Cutoff > 6.50 30	78.30	86.21	95.40	52.08	80.00
Cutoff > 6.70 6	77.36	86.21	95.35	51.02	79.26
**Validation cohort (n = 58)**					
Cutoff > 5.30 and metastases	88.10	81.25	92.50	72.22	86.21
Cutoff > 5.30	83.33	81.25	92.11	65.00	82.76
Cutoff > 3.20 11, 29	100.0	18.75	72.41	100.00	77.59
Cutoff > 4.00 14, 31	90.47	50.00	82.61	66.67	79.31
Cutoff > 6.50 30	76.19	81.25	91.43	56.52	77.59
Cutoff > 6.70 6	71.42	81.25	90.91	52.00	74.14

## Abbreviations

SUV_max_: Maximum standardized uptake value
csPCa: clinically significant prostate cancer
cnsPCa: clinically non-significant prostate cancer
BPD: benign prostatic diseases
BPH: benign prostate hypertrophy
PCa: prostate cancer
DRE: digital rectal examination
tPSA: total prostate-specific antigen
mpMRI: multi-parameter MRI
GS: Gleason score
ROC: Receiver operating characteristic
^68^Ga-PSMA: ^68^Ga-labeled prostate-specific antigen
VOI: volume of interest
ADT: deprivation treatment
RP: radical prostatectomy
RT: radiotherapy
LNMs: lymph node metastases
BMs: bone metastases
cnsPCa: clinically non-significant PCa
EANM: the Joint European Association of Nuclear Medicine
SNMMI: Society of Nuclear Medicine and Molecular Imaging
TRUS: transrectal ultrasound
HE: hematoxylin-eosin
IHC: immunohistochemistry
AJCC: American Joint Committee on Cancer
ICC: Intraclass correlation co-efficient
lcsPCa: localised csPCa

## References

[1] Sung H, Ferlay J, Siegel RL, Laversanne M, Soerjomataram I, Jemal A (2021). Global cancer statistics 2020: GLOBOCAN estimates of incidence and mortality worldwide for 36 cancers in 185 countries. CA Cancer J Clin.

[2] Klotz L, Vesprini D, Sethukavalan P, Jethava V, Zhang L, Jain S (2015). Long-term follow-up of a large active surveillance cohort of patients with prostate cancer. J Clin Oncol.

[3] Epstein JI, Egevad L, Amin MB, Delahunt B, Srigley JR, Humphrey PA (2016). The 2014 International Society of Urological Pathology (ISUP) Consensus Conference on Gleason Grading of Prostatic Carcinoma: Definition of Grading Patterns and Proposal for a New Grading System. Am J Surg Pathol.

[4] Mottet N, Bellmunt J, Bolla M, Briers E, Cumberbatch MG, De Santis M (2017). EAU-ESTRO-SIOG Guidelines on Prostate Cancer. Part 1: Screening, Diagnosis, and Local Treatment with Curative Intent. Eur Urol.

[5] Mohler JL, Antonarakis ES, Armstrong AJ, D'Amico AV, Davis BJ, Dorff T (2019). Prostate Cancer, Version 2.2019, NCCN Clinical Practice Guidelines in Oncology. J Natl Compr Canc Netw.

[6] Hicks RM, Simko JP, Westphalen AC, Nguyen HG, Greene KL, Zhang L (2018). Diagnostic Accuracy of (68)Ga-PSMA-11 PET/MRI Compared with Multiparametric MRI in the Detection of Prostate Cancer. Radiology.

[7] Giesel FL, Sterzing F, Schlemmer HP, Holland-Letz T, Mier W, Rius M (2016). Intra-individual comparison of (68)Ga-PSMA-11-PET/CT and multi-parametric MR for imaging of primary prostate cancer. Eur J Nucl Med Mol Imaging.

[8] Hofman MS, Lawrentschuk N, Francis RJ, Tang C, Vela I, Thomas P (2020). Prostate-specific membrane antigen PET-CT in patients with high-risk prostate cancer before curative-intent surgery or radiotherapy (proPSMA): a prospective, randomised, multicentre study. Lancet.

[9] Fanti S, Goffin K, Hadaschik BA, Herrmann K, Maurer T, MacLennan S (2021). Consensus statements on PSMA PET/CT response assessment criteria in prostate cancer. Eur J Nucl Med Mol Imaging.

[10] Afshar-Oromieh A, Malcher A, Eder M, Eisenhut M, Linhart HG, Hadaschik BA (2013). PET imaging with a [68Ga]gallium-labelled PSMA ligand for the diagnosis of prostate cancer: biodistribution in humans and first evaluation of tumour lesions. Eur J Nucl Med Mol Imaging.

[11] Woythal N, Arsenic R, Kempkensteffen C, Miller K, Janssen JC, Huang K (2018). Immunohistochemical Validation of PSMA Expression Measured by (68)Ga-PSMA PET/CT in Primary Prostate Cancer. Journal of nuclear medicine: official publication, Society of Nuclear Medicine.

[12] Bostwick DG, Pacelli A, Blute M, Roche P, Murphy GP (1998). Prostate specific membrane antigen expression in prostatic intraepithelial neoplasia and adenocarcinoma: a study of 184 cases. Cancer.

[13] Rahbar K, Weckesser M, Huss S, Semjonow A, Breyholz HJ, Schrader AJ (2016). Correlation of Intraprostatic Tumor Extent with (6)(8)Ga-PSMA Distribution in Patients with Prostate Cancer. Journal of nuclear medicine: official publication, Society of Nuclear Medicine.

[14] Demirci E, Kabasakal L, Sahin OE, Akgun E, Gultekin MH, Doganca T (2019). Can SUVmax values of Ga-68-PSMA PET/CT scan predict the clinically significant prostate cancer?. Nucl Med Commun.

[15] Uprimny C, Kroiss AS, Decristoforo C, Fritz J, von Guggenberg E, Kendler D (2017). (68)Ga-PSMA-11 PET/CT in primary staging of prostate cancer: PSA and Gleason score predict the intensity of tracer accumulation in the primary tumour. Eur J Nucl Med Mol Imaging.

[16] Roberts MJ, Morton A, Donato P, Kyle S, Pattison DA, Thomas P (2021). (68)Ga-PSMA PET/CT tumour intensity pre-operatively predicts adverse pathological outcomes and progression-free survival in localised prostate cancer. Eur J Nucl Med Mol Imaging.

[17] Donato P, Roberts MJ, Morton A, Kyle S, Coughlin G, Esler R (2019). Improved specificity with (68)Ga PSMA PET/CT to detect clinically significant lesions "invisible" on multiparametric MRI of the prostate: a single institution comparative analysis with radical prostatectomy histology. Eur J Nucl Med Mol Imaging.

[18] Fendler WP, Eiber M, Beheshti M, Bomanji J, Ceci F, Cho S (2017). (68)Ga-PSMA PET/CT: Joint EANM and SNMMI procedure guideline for prostate cancer imaging: version 1.0. Eur J Nucl Med Mol Imaging.

[19] Rauscher I, Maurer T, Fendler WP, Sommer WH, Schwaiger M, Eiber M (2016). (68)Ga-PSMA ligand PET/CT in patients with prostate cancer: How we review and report. Cancer Imaging.

[20] Souvatzoglou M, Weirich G, Schwarzenboeck S, Maurer T, Schuster T, Bundschuh RA (2011). The sensitivity of [11C]choline PET/CT to localize prostate cancer depends on the tumor configuration. Clin Cancer Res.

[21] Edge SB, Compton CC (2010). The American Joint Committee on Cancer: the 7th edition of the AJCC cancer staging manual and the future of TNM. Ann Surg Oncol.

[22] Zhang J, Shao S, Wu P, Liu D, Yang B, Han D (2019). Diagnostic performance of (68)Ga-PSMA PET/CT in the detection of prostate cancer prior to initial biopsy: comparison with cancer-predicting nomograms. Eur J Nucl Med Mol Imaging.

[23] Jiao D, Li Y, Yang F, Han D, Wu J, Shi S (2019). Expression of Prostate-Specific Membrane Antigen in Tumor-Associated Vasculature Predicts Poor Prognosis in Hepatocellular Carcinoma. Clin Transl Gastroenterol.

[24] Ferraro DA, Ruschoff JH, Muehlematter UJ, Kranzbuhler B, Muller J, Messerli M (2020). Immunohistochemical PSMA expression patterns of primary prostate cancer tissue are associated with the detection rate of biochemical recurrence with (68)Ga-PSMA-11-PET. Theranostics.

[25] Paschalis A, Sheehan B, Riisnaes R, Rodrigues DN, Gurel B, Bertan C (2019). Prostate-specific Membrane Antigen Heterogeneity and DNA Repair Defects in Prostate Cancer. Eur Urol.

[26] Hirsch FR, Varella-Garcia M, Bunn PA Jr, Di Maria MV, Veve R, Bremmes RM (2003). Epidermal growth factor receptor in non-small-cell lung carcinomas: correlation between gene copy number and protein expression and impact on prognosis. J Clin Oncol.

[27] Domachevsky L, Goldberg N, Bernstine H, Nidam M, Groshar D (2018). Quantitative characterisation of clinically significant intra-prostatic cancer by prostate-specific membrane antigen (PSMA) expression and cell density on PSMA-11. Eur Radiol.

[28] Mottet N, van den Bergh RCN, Briers E, Van den Broeck T, Cumberbatch MG, De Santis M (2021). EAU-EANM-ESTRO-ESUR-SIOG Guidelines on Prostate Cancer-2020 Update. Part 1: Screening, Diagnosis, and Local Treatment with Curative Intent. Eur Urol.

[29] Prasad V, Steffen IG, Diederichs G, Makowski MR, Wust P, Brenner W (2016). Biodistribution of [(68)Ga]PSMA-HBED-CC in Patients with Prostate Cancer: Characterization of Uptake in Normal Organs and Tumour Lesions. Mol Imaging Biol.

[30] Fendler WP, Schmidt DF, Wenter V, Thierfelder KM, Zach C, Stief C (2016). 68Ga-PSMA PET/CT Detects the Location and Extent of Primary Prostate Cancer. Journal of nuclear medicine: official publication, Society of Nuclear Medicine.

[31] Ferraro DA, Muehlematter UJ, Garcia Schuler HI, Rupp NJ, Huellner M, Messerli M (2019). (68)Ga-PSMA-11 PET has the potential to improve patient selection for extended pelvic lymph node dissection in intermediate to high-risk prostate cancer. Eur J Nucl Med Mol Imaging.

[32] Silver DA, Pellicer I, Fair WR, Heston WD, Cordon-Cardo C (1997). Prostate-specific membrane antigen expression in normal and malignant human tissues. Clin Cancer Res.

[33] Wright GL Jr, Haley C, Beckett ML, Schellhammer PF (1995). Expression of prostate-specific membrane antigen in normal, benign, and malignant prostate tissues. Urol Oncol.

[34] Sweat SD, Pacelli A, Murphy GP, Bostwick DG (1998). Prostate-specific membrane antigen expression is greatest in prostate adenocarcinoma and lymph node metastases. Urology.

[35] Gupta M, Choudhury PS, Rawal S, Gupta G (2018). Incremental value of 68-gallium-prostate-specific membrane antigen positron emission tomography/computed tomography in patients with abnormal prostate-specific antigen and benign transrectal ultrasound biopsy. Urol Ann.

[36] Demirci E, Sahin OE, Ocak M, Akovali B, Nematyazar J, Kabasakal L (2016). Normal distribution pattern and physiological variants of 68Ga-PSMA-11 PET/CT imaging. Nucl Med Commun.

[37] Sachpekidis C, Kopka K, Eder M, Hadaschik BA, Freitag MT, Pan L (2016). 68Ga-PSMA-11 Dynamic PET/CT Imaging in Primary Prostate Cancer. Clin Nucl Med.

[38] Murphy GP, Elgamal AA, Su SL, Bostwick DG, Holmes EH (1998). Current evaluation of the tissue localization and diagnostic utility of prostate specific membrane antigen. Cancer.

[39] Ghosh A, Heston WD (2004). Tumor target prostate specific membrane antigen (PSMA) and its regulation in prostate cancer. J Cell Biochem.

[40] Ferraro DA, Rupp NJ, Donati OF, Messerli M, Eberli D, Burger IA (2019). 68Ga-PSMA-11 PET/MR Can Be False Positive in Normal Prostatic Tissue. Clin Nucl Med.

[41] Scheltema MJ, Chang JI, Stricker PD, van Leeuwen PJ, Nguyen QA, Ho B (2019). Diagnostic accuracy of (68) Ga-prostate-specific membrane antigen (PSMA) positron-emission tomography (PET) and multiparametric (mp)MRI to detect intermediate-grade intra-prostatic prostate cancer using whole-mount pathology: impact of the addition of (68) Ga-PSMA PET to mpMRI. BJU Int.

[42] Liu C, Liu T, Zhang Z, Zhang N, Du P, Yang Y (2020). (68)Ga-PSMA PET/CT Combined with PET/Ultrasound-Guided Prostate Biopsy Can Diagnose Clinically Significant Prostate Cancer in Men with Previous Negative Biopsy Results. Journal of nuclear medicine: official publication, Society of Nuclear Medicine.

[43] Perera M, Papa N, Roberts M, Williams M, Udovicich C, Vela I (2020). Gallium-68 Prostate-specific Membrane Antigen Positron Emission Tomography in Advanced Prostate Cancer-Updated Diagnostic Utility, Sensitivity, Specificity, and Distribution of Prostate-specific Membrane Antigen-avid Lesions: A Systematic Review and Meta-analysis. Eur Urol.

[44] Yin H, Chen M, Qiu X, Qiu L, Gao J, Li D (2021). Can (68)Ga-PSMA-11 PET/CT predict pathological upgrading of prostate cancer from MRI-targeted biopsy to radical prostatectomy?. Eur J Nucl Med Mol Imaging.

[45] Todenhofer T, Gratzke C (2020). Re: Prostate-specific Membrane Antigen Heterogeneity and DNA Repair Defects in Prostate Cancer. Eur Urol.

[46] Gao J, Zhang C, Zhang Q, Fu Y, Zhao X, Chen M (2019). Diagnostic performance of (68)Ga-PSMA PET/CT for identification of aggressive cribriform morphology in prostate cancer with whole-mount sections. Eur J Nucl Med Mol Imaging.

[47] Ceci F, Oprea-Lager DE, Emmett L, Adam JA, Bomanji J, Czernin J, et al. E-PSMA: the EANM standardized reporting guidelines v1.0 for PSMA-PET. Eur J Nucl Med Mol Imaging. 2021 10.1007/s00259-021-05245-y PMC8113168 33604691

[48] Giesel FL, Hadaschik B, Cardinale J, Radtke J, Vinsensia M, Lehnert W (2017). F-18 labelled PSMA-1007: biodistribution, radiation dosimetry and histopathological validation of tumor lesions in prostate cancer patients. Eur J Nucl Med Mol Imaging.

[49] Cysouw MCF, Jansen BHE, van de Brug T, Oprea-Lager DE, Pfaehler E, de Vries BM (2021). Machine learning-based analysis of [(18)F]DCFPyL PET radiomics for risk stratification in primary prostate cancer. Eur J Nucl Med Mol Imaging.

[50] Zamboglou C, Carles M, Fechter T, Kiefer S, Reichel K, Fassbender TF (2019). Radiomic features from PSMA PET for non-invasive intraprostatic tumor discrimination and characterization in patients with intermediate- and high-risk prostate cancer - a comparison study with histology reference. Theranostics.

[51] Zhao J, Hamm B, Brenner W, Makowski MR (2020). Lesion-to-background ratio threshold value of SUVmax of simultaneous [(68)Ga]Ga-PSMA-11 PET/MRI imaging in patients with prostate cancer. Insights Imaging.

