# Supplementary material for: Establishment and prospective validation of an SUVmax cutoff value to discriminate clinically significant prostate cancer from benign prostate diseases in patients with suspected prostate cancer by 68Ga-PSMA PET/CT: a real-world study
Source: Theranostics. 2021 Jul 25;11(17):8396–411. doi: 10.7150/thno.58140 (PMC8344003; doi:10.7150/thno.58140)
Supplement: Supplementary file 1 — Supplementary methods, figures and tables. [file thnov11p8396s1.pdf]

Supplementary Methods

Methods

Collection and evaluation of ^{68}Ga-PSMA PET/CT images

PSMA N,N'-bis [2-hydroxy-5-(carboxyethyl)benzyl] ethylenediamine-N,N'-diacetic acid (PSMA-HBED-CC) was acquired from ABX GmbH (Radeberg, Germany), and the 68Ga/68Ge generator system was obtained from ITG GmbH (Munich, Germany). PSMA-HBED-CC was labeled with ^{68}Ga as we previously reported[14], and the patients were intravenously injected with 1.80-2.20 MBq/kg body weight ^{68}Ga-PSMA-11. Low-dose CT (pitch 0.8, 50 mA, 120 kV[peak]) scans for PET attenuation were acquired (automatic mA, 120 keV, 512x512 matrix, 5-mm slice thickness, 1.0-s rotation time, and 0.8 pitch), followed by a PET scan with 5 bed positions (3 minutes/bed, from the head to the proximal thighs) performed approximately sixty minutes after tracer injection. The PET/CT images were then transferred to a multimodal workstation for data analysis (Syngo Truepoint Siemens Medical Solutions).

IHC staining and evaluation

The dominant staining intensity (0=negative, 1=weak, 2=moderate, 3=strong, and 4=extremely strong) and percentage of positive cells (0% to 100%) were evaluated and multiplied to assess the H-score (Figure S1). In hence, the overall score ranged from 0.00 to 400.00.

Intraclass correlation coefficient analysis

ICC results were evaluated as follows: 0.00-0.20 suggests poor agreement; 0.20-0.40 suggests fair agreement; 0.40-0.60 suggests moderate agreement; 0.60-0.80 suggests good agreement; greater than 0.80 suggests very good agreement.

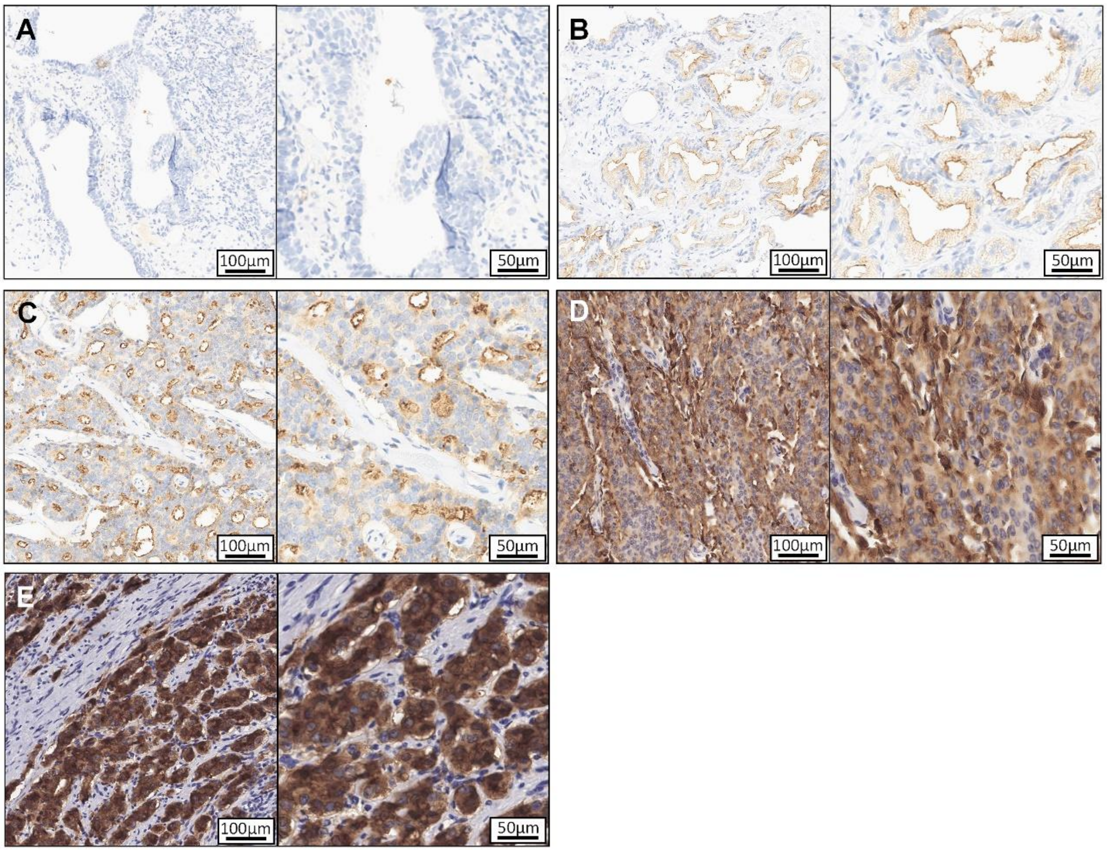

Figure S1. Representative IHC staining results for PSMA showing the staining intensity classifications: negative (A), weak (B), moderate (C), strong (D), and extremely strong (E). IHC staining was performed with a monoclonal anti-PSMA antibody (clone 1D6, 1:100, MAB-0672, MXB Biotechnologies).

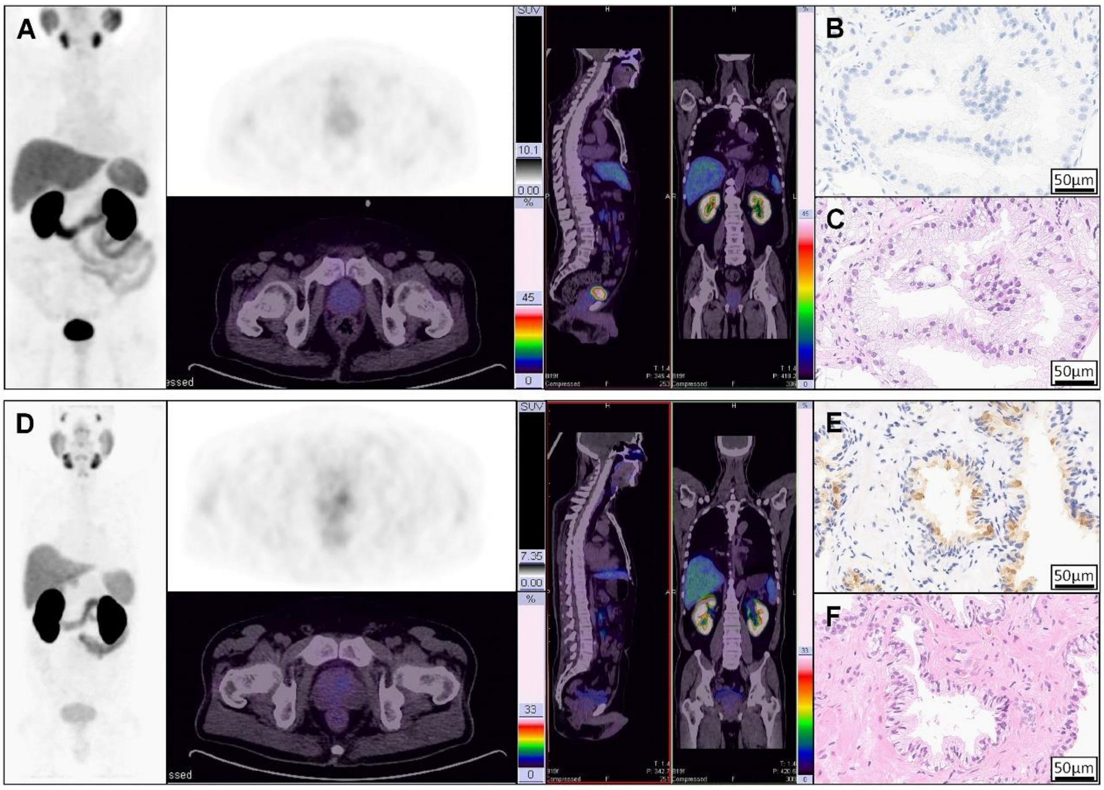

Figure S2. False negative and false positive results of ^{68}Ga-PSMA PET/CT. ^{68}Ga-PSMA PET/CT images (A, D), PSMA staining results (B, E), and HE staining results (C, F) for a patient pathologically diagnosed with PCa (A–C) and a patient pathologically diagnosed with BPD (D–F). The patient with PCa was negative by ^{68}Ga-PSMA PET/CT (A, SUV_{max} = 3.10) and PSMA staining (B). The patient with BPD was positive by ^{68}Ga-PSMA PET/CT (D, SUV_{max} = 3.90) and PSMA staining (E) but negative by HE staining (F). The results from PSMA PET/CT were in consensus with those from IHC staining. The results from HE staining were evaluated by pathologists and used as a reference.

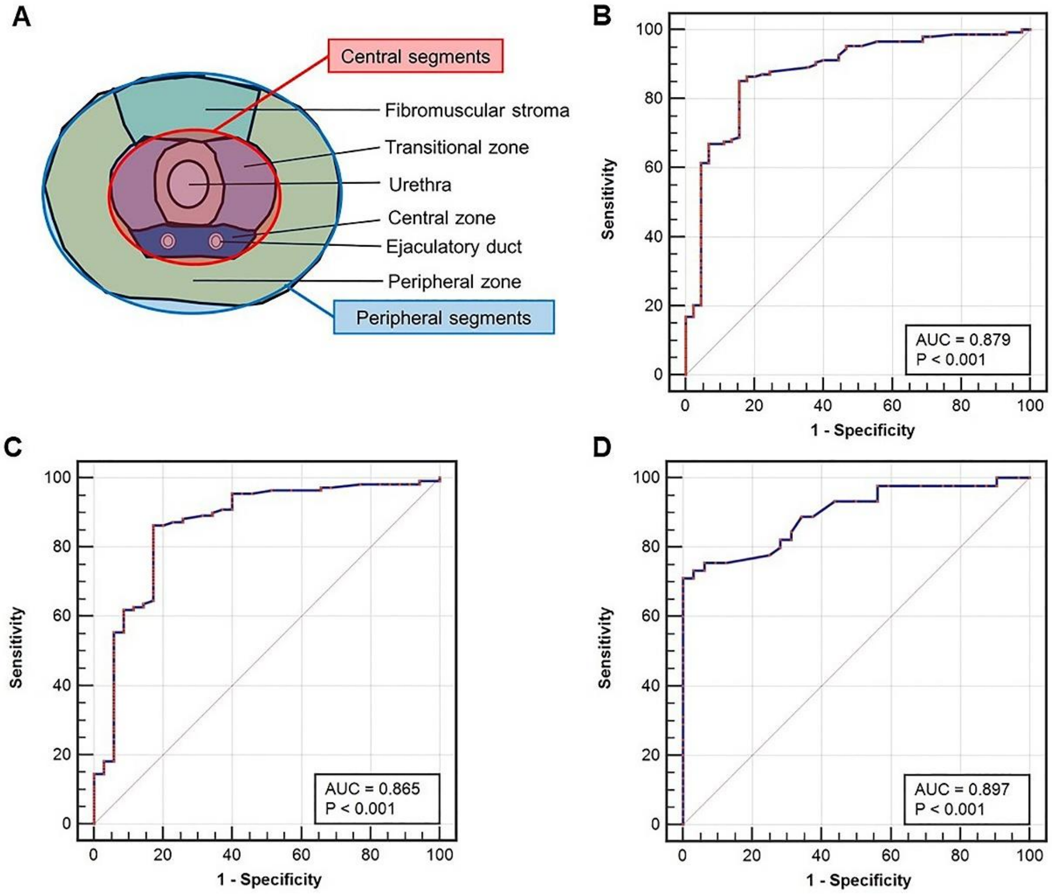

Figure S3. ROC curves in zonal anatomy analysis. (A) Anatomical structure of the prostate. (B) ROC curve for diagnosing csPCa in all patients. The SUV_{max} value of 5.30 was the best cutoff for diagnosing csPCa. (C) ROC curve for diagnosing peripheral csPCa in patients with negative PSMA PET results and patients with PSMA uptake in peripheral segments only. The SUV_{max} value of 5.30 was the best cutoff for diagnosing peripheral csPCa. (D) ROC curve for diagnosing central csPCa in patients with negative PSMA PET results and patients with PSMA uptake in the central segments. The SUV_{max} value of 9.00 was the best cutoff for diagnosing central csPCa. The SUV_{max} cutoff value for diagnosing central csPCa was higher than that for diagnosing peripheral csPCa. The top and

bottom ROC curves represent the upper and lower bounds of the 95% confidence interval of the middle bound, respectively. The peripheral and central segments were delineated based on a previous study [20].

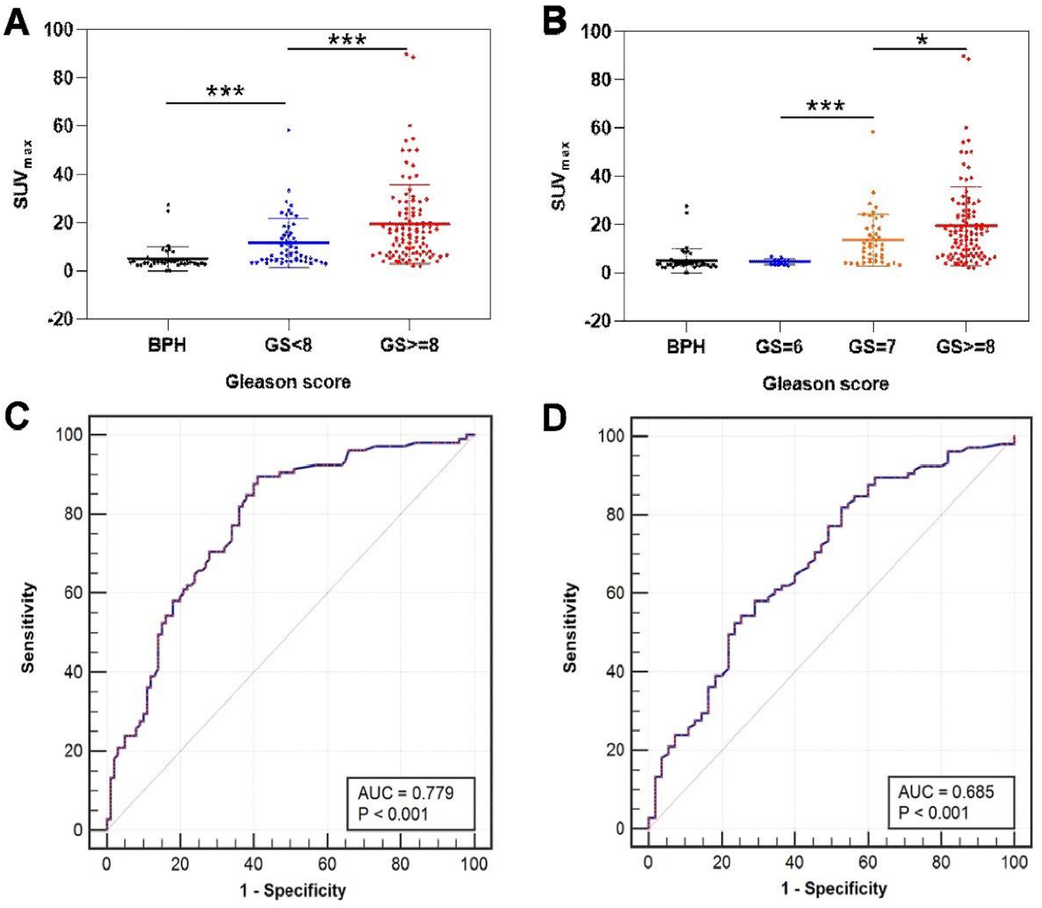

Figure S4. Comparison of SUV_{max} values for diagnosing patients with high-risk PCa among all patients and patients with PCa. (A) The SUV_{max} values of the GS < 8 group were significantly higher than those of the BPD group, but significantly lower than those of the GS ≥ 8 group. (B) The SUV_{max} values of the GS = 7 group were significantly higher than those of the GS = 6 group, but lower than those of the GS ≥ 8 group. (C) ROC curve showing that the best cutoff value was 5.30 for diagnosing patients with high-risk PCa among all patients (PCa or BPD). (D) ROC curve showing that the best cutoff was 6.50 for diagnosing patients with high-risk PCa among all patients with PCa. (*, $P < 0.05$; **, $P < 0.01$; ***, $P < 0.001$).

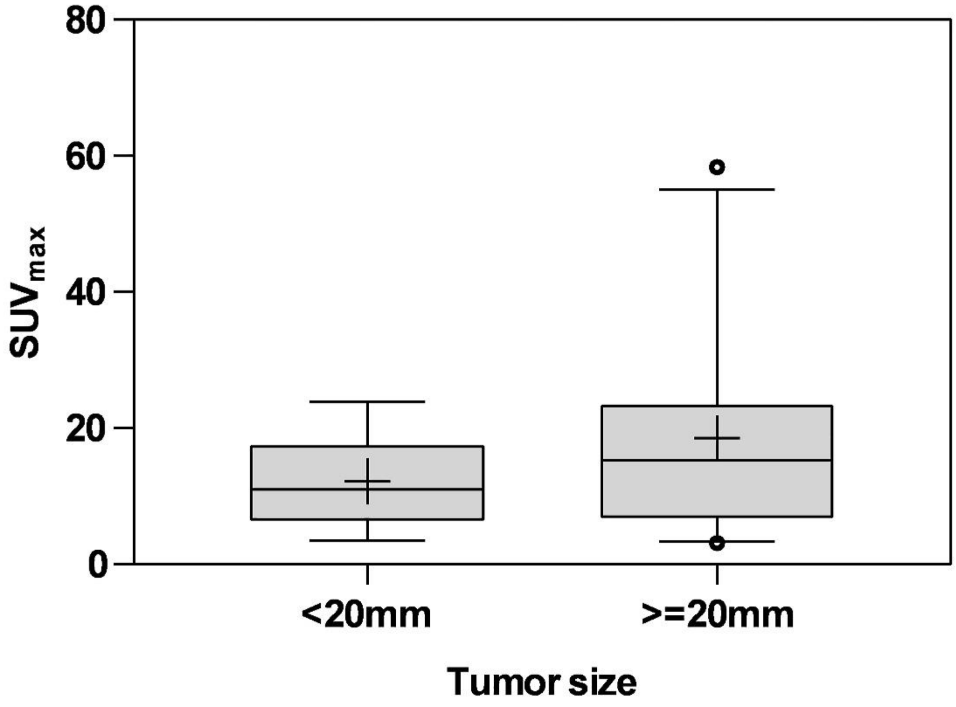

Figure S5. Box plots comparing the SUV_{max} values across PCa tumor sizes (n = 48). The vertical borders of the box represent the 25th and 75th percentiles, the middle bar represents the median, and "+" represents the mean. The error bars represent the 5th and 95th percentiles, and the circles represent outliers. The mean SUV_{max} value was 10.55 ± 6.34 in primary PCa tumors with a diameter < 20 mm (n = 24) and 18.47 ± 14.49 in tumors with a diameter ≥ 20 mm (n = 24, Mann-Whitney U test, P = 0.061). SUV_{max} is weakly correlated with tumor size (Spearman's ρ, r_s = 0.332, P < 0.001).

Table S1. Pathological diagnosis of patients with BPD.

Pathological diagnosis							n (%)
BPH	Chronic prostatitis	Acute prostatitis	Atrophy	Necrosis	Calcification	Interstitial hypertrophy	
+	+	-	-	-	-	+	6 (13.3)
+	+	+	-	-	-	+	4 (8.9)
+	+	-	-	+	+	+	1 (2.2)
+	+	+	-	-	-	-	3 (6.7)
+	+	-	+	-	-	-	1 (2.2)
+	+	-	-	-	-	-	30 (66.7)

Table S2. Characteristics of patients with BPD or lcsPCa.

Characteristic	Training cohort (n = 75)				Validation cohort (n = 37)			
	BPD	lcsPCa	*χ/z*	*P*	BPD	lcsPCa	*χ/z*	*P*
**n (%)**	29 (38.7)	46 (61.3)	——	——	16 (43.2)	21 (56.8)	——	——
**Mean age, y**	68.21 ± 9.37	70.46 ± 7.67	——	0.295	64.56 ± 10.83	68.90 ± 10.54	——	0.354
**Mean acquisition time, min after injection**	61.79 ± 9.90	65.24 ± 13.94	——	0.427	65.56 ± 12.64	68.29 ± 13.08	——	0.439
**Mean interval between biopsy and PSMA PET/CT, d**	10.04 ± 6.15	10.54 ± 7.03	——	0.198	10.07 ± 6.64	9.81 ± 7.17	——	0.639
**Mean H-score**	41.44 ± 39.93	177.71 ± 97.36	–5.876	<0.001*	59.25 ± 85.17	156.00 ± 97.00	–3.11	<0.001*
**Median tPSA, ng/mL (P_{25}–P_{75})**	11.73 (7.10–14.90)	15.88 (8.67–35.50)	–1.697	<0.001*	9.72 (7.46–13.56)	18.16 (110.09–19.44)	–2.36	<0.001*
≤4, n (%)	4/75 (5.3)	8/75 (10.7)	——	——	1/37 (2.7)	1/37 (2.7)	——	——
4–10, n (%)	6/75 (8.0)	8/75 (10.7)	——	——	8/37 (1.6)	4/37 (10.8)	——	——
10–20, n (%)	14/75 (18.7)	13/75 (17.3)	——	——	5/37 (13.5)	6/37 (16.2)	——	——
>20, n (%)	5/75 (6.7)	17/75 (22.7)	——	——	2/37 (5.4)	10/37 (27.0)	——	——
**GS, n (%)**	——	46 (100.0)	——	——	——	21 (100.0)	——	——
7 (3 + 4)	——	7/46 (15.2)	——	——	——	7/21 (33.3)	——	——
7 (4 + 3)	——	13/46 (28.3)	——	——	——	5/42 (23.8)	——	——
8 (4 + 4)	——	18/46 (39.1)	——	——	——	9/21 (42.9)	——	——
8 (5 + 3)	——	1/46 (2.2)	——	——	——	0/21 (0.0)	——	——
9 (4 + 5)	——	4/46 (8.7)	——	——	——	0/21 (0.0)	——	——
9 (5 + 4)	——	3/46 (6.5)	——	——	——	0/21 (0.0)	——	——

10 (5 + 5)	——	0/46 (0.0)	——	——	——	0/21 (0.0)	——	——

* Statistically significant; average age, acquisition time, and interval were compared using independent samples *t*-tests; average H-score and tPSA were compared using Wilcoxon W tests.

Mean values are presented as mean ± SD.

Table S3. Comparison of patient characteristics between the training and validation cohorts.

Characteristic	All patients				Patients without metastases			
	Training cohort	Validation cohort	χ/z	*P*	Training cohort	Validation cohort	χ/z	*P*
**n (%)**	135 (69.9)	58 (30.1)	——	——	75 (67.0)	37 (33.0)	——	——
**Mean age, y**	69.74 ± 8.52	68.90 ± 10.30	——	0.472	69.59 ± 8.38	67.03 ± 10.74	——	0.105
**Mean acquisition time, min after injection**	64.96 ± 12.80	65.60 ± 12.99	——	0.223	63.91 ± 12.63	67.11 ± 12.79	——	0.095
**Mean interval between biopsy and PSMA PET/CT, d**	10.75 ± 5.66	9.90 ± 2.22	——	0.815	10.19 ± 6.61	10.08 ± 6.71	——	0.901
**Mean H-score**	165.97 ± 113.62	140.95 ± 109.30	– 5.876	0.596	125.02 ± 104.17	114.16 ± 103.00	– 0.520	0.603
**Median tPSA, ng/mL (P_{25}–P_{75})**	19.09 (8.68–123.30)	18.98 (9.49–50.39)	– 1.224	0.221	13.00 (8.04–26.05)	13.64 (8.47–25.40)	– 0.331	0.741
≤4, n (%)	22/135 (16.3)	3/58 (5.2)	——	——	12/75 (16.0)	2/37 (5.4)	——	——
4–10, n (%)	15/135 (11.1)	13/58 (22.4)	——	——	14/75 (18.7)	12/37 (32.4)	——	——
10–20, n (%)	31/135 (23.0)	14/58 (24.1)	——	——	27/75 (36.0)	11/37 (29.7)	——	——
>20, n (%)	67/135 (49.6)	28/58 (48.3)	——	——	22/75 (29.3)	12/37 (32.4)	——	——
**GS, n (%)**	106 (100.0）	42 (100.0)	——	——	46 (100.0)	21 (100.0)	——	——
7 (3 + 4)	11/106 (10.4)	7/42 (16.7)	——	——	7/46 (15.2)	7/21 (33.3)	——	——
7 (4 + 3)	21/106 (19.8)	5/42 (11.9)	——	——	13/46 (28.3)	5/42 (23.8)	——	——
8 (4 + 4)	41/106 (38.7)	14/42 (33.3)	——	——	18/46 (39.1)	9/21 (42.9)	——	——
8 (5 + 3)	3/106 (2.8)	1/42 (2.4)	——	——	1/46 (2.2)	0/21 (0.0)	——	——
9 (4 + 5)	14/106 (13.2)	9/42 (21.4)	——	——	4/46 (8.7)	0/21 (0.0)	——	——
9 (5 + 4)	9/106 (8.5)	2/42 (4.8)	——	——	3/46 (6.5)	0/21 (0.0)	——	——
10 (5 + 5)	7/106 (6.6)	4/42 (9.5)	——	——	0/46 (0.0)	0/21 (0.0)	——	——

* Statistically significant; average age, acquisition time, and interval were compared using independent samples *t*-tests; average H-score and tPSA were compared using Wilcoxon W tests.

Mean values are presented as mean ± SD.

Table S4A. PSMA expression in patients with BPD or csPCa.

Groups	H-score		Comparison	*P*
	Mean ± SD	Median (P_{25}–P_{75})		
**Diagnosis**				
BPH (n = 45)	45.41 ± 60.17	24.00 (12.50–57.00)	BPH vs. lcsPCa	<0.001*
lcsPCa (n = 67)	177.10 ± 98.56	188.00 (99.00–243.00)	lcsPCa vs. mcsPCa	0.095
mcsPCa (n = 81)	205.83 ± 103.43	204.00 (126.75–306.00)	BPH vs. mcsPCa	<0.001*

* Statistically significant, Mann-Whitney U test

Table S4B. SUV_{max} values in patients with BPD or csPCa.

Groups	SUV_{max}		Comparison	*P*
	Mean ± SD	Median (P_{25}–P_{75})		
**Diagnosis**				
BPH (n = 45)	5.03 ± 5.04	3.64 (3.10–4.43)	BPH vs. lcsPCa	<0.001*
lcsPCa (n = 67)	13.65 ± 9.87	11.30 (6.20–19.20)	lcsPCa vs. mcsPCa	0.005
mcsPCa (n = 81)	20.94 ± 17.63	17.03 (8.05–25.6)	BPH vs. mcsPCa	<0.001*
**H-score**				
0–75 (n = 61)	5.10 ± 5.33	4.00 (3.35–4.90)	0–75 vs. 76–150	<0.001*
76–150 (n = 30)	7.53 ± 4.50	6.54 (4.48–8.07)	76–150 vs. 151–225	<0.001*
151–225 (n = 49)	14.41 ± 6.16	13.51 (10.10–18.35)	151–225 vs. >225	<0.001*
>225 (n = 53)	30.08 ± 17.56	25.00 (17.80–36.30)	0–75 vs. >225	<0.001*
**Intensity of staining**				
0–1 (n = 35)	5.54 ± 6.94	4.00 (3.10–4.46)	0–1 vs. 2	0.017*
2 (n = 44)	6.48 ± 4.51	4.95 (3.50–7.61)	2 vs. 3	<0.001*
3 (n = 51)	13.05 ± 6.62	12.40 (7.40–18.40)	3 vs. 4	<0.001*
4 (n = 63)	26.87 ± 17.86	22.60 (16.00–31.80)	0–1 vs. 4	<0.001*
**Percentage of stained cells (%)**				
0–25 (n = 43)	5.43 ± 6.25	3.90 (3.20–4.40)	0–25 vs. 26–50	0.011*
26–50 (n = 44)	6.43 ± 4.11	5.46 (3.61–7.70)	26–50 vs. 51–75	<0.001*
51–75 (n = 59)	14.98 ± 7.60	13.51 (9.60–19.40)	51–75 vs. 76–100	<0.001*

76–100 (n = 47)	30.57 ± 18.41	25.00 (17.20–39.40)	0–25 vs. 76–100	<0.001*

* Statistically significant, Mann-Whitney U test

Table S4C. Percentage of stained cells in comparison to intensity of PSMA IHC staining.

Groups	Percentage of stained cells		Comparison	*P*
	Mean ± SD	Median (P_{25}–P_{75})		
**Intensity of staining**				
0–1 (n = 35)	18.14 ± 16.39	14.00 (4.00–26.00)	0–1 vs. 2	<0.001
2 (n = 44)	38.27 ± 21.53	34.00 (23.25–53.25)	2 vs. 3	<0.001
3 (n = 51)	58.61 ± 17.95	61.00 (48.00–75.00)	3 vs. 4	0.003
4 (n = 63)	69.19 ± 18.88	76.00 (52.00–84.00)	0–1 vs. 4	<0.001

* Statistically significant, Mann-Whitney U test

Table S5A. H-scores and SUV_{max} values in GS groups.

GS	H-score		SUV_{max}	
	Mean ± SD	Median (P_{25}–P_{75})	Mean ± SD	Median (P_{25}–P_{75})
**0 (n = 45)**	——	——	——	——
0 (n = 45)	47.78 ± 59.99	28.00 (12.50–58.00)	5.03 ± 5.04	3.64 (3.10–4.43)
**6 (n = 12)**	——	——	——	——
3 + 3 (n = 12)	53.75 ± 38.41	52.00 (18.00–87.50)	4.43 ± 1.22	4.35 (3.33–5.25)
**7 (n = 43)**	177.63 ± 105.20	177.00 (100.00–272.00)	13.51 ± 10.66	11.40 (5.10–18.40)
3 + 4 (n = 17)	148.29 ± 90.99	159.00 (79.00–207.50)	9.98 ± 5.75	8.57 (5.00–13.46)
4 + 3 (n = 26)	196.81 ± 111.02	192.75 (102.50–308.00)	15.82 ± 12.49	13.45 (4.93–23.85)
**8 (n = 59)**	208.29 ± 106.46	220.50 (117.50–304.00)	19.98 ± 18.57	16.20 (7.20–25.30)
4 + 4 (n = 55)	205.03 ± 108.54	212.00 (114.00–304.00)	19.90 ± 19.13	16.00 (6.90–25.30)
5+3 (n = 4)	253.13 ± 63.94	254.25 (190.13–315.00)	21.03 ± 8.64	18.37 (14.68–30.05)
**9 (n = 35)**	186.07 ± 79.99	192.00 (145.00–225.00)	16.94 ± 10.32	15.80 (10.10–20.50)
4 + 5 (n = 25)	177.74 ± 80.94	189.00 (129.75–224.50)	15.50 ± 10.82	12.10 (9.05–18.30)
5 + 4 (n = 10)	206.90 ± 77.63	208.00 (169.50–231.75)	20.56 ± 8.35	19.38 (16.60–24.25)
**10 (n = 11)**	——	——	——	——
5 + 5 (n = 11)	181.09 ± 138.68	159.00 (26.00–324.00)	23.50 ± 18.49	23.30 (5.74–43.80)

Table S5B. Comparison of H-scores and SUV_{max} values in GS groups.

GS	***P*** **(H-score)**	***P*** **(SUV_{max})**
0 vs. 3 + 3	0.256	0.337
0 vs. 3 + 4	<0.001*	<0.001*
3 + 3 vs. 3 + 4	0.006	0.001*
3 + 4 vs. 4 + 3	0.160	0.180
4 + 3 vs. 4 + 4	0.716	0.501
4 + 4 vs. 5 + 3	0.493	0.356
4 + 4 vs. 4 + 5	0.142	0.124
4 + 5 vs. 5 + 4	0.521	0.041*
5 + 4 vs. 5 + 5	0.387	0.973
0 vs. 7	<0.001*	<0.001*
6 vs. 7	<0.001*	<0.001*
7 vs. 8	0.141	0.066
8 vs. 9	0.253	0.953
9 vs. 10	0.559	0.648

* Statistically significant; Mann-Whitney U test.

Table S6. Sensitivity, specificity, PPV, NPV, and accuracy of ^{68}Ga-PSMA PET/CT in detecting lcsPCa.

	Sensitivity (%)	Specificity (%)	PPV (%)	NPV (%)	Accuracy (%)
**All patients (BPD or lcsPCa, n = 112)**					
Cutoff > 5.30	79.10	84.44	88.33	73.08	81.25
Cutoff > 3.20 [11, 29]	97.01	31.11	67.71	87.50	70.54
Cutoff > 4.00 [14, 31]	83.58	55.56	69.44	73.68	72.32
Cutoff > 6.50 [30]	70.15	84.44	87.04	65.52	75.89
Cutoff > 6.70 [6]	68.66	84.44	86.79	64.41	75.00
**Training cohort (n = 75)**					
Cutoff > 5.30	80.43	86.21	90.24	73.53	82.67
Cutoff > 3.20 [11, 29]	95.65	37.93	70.97	84.62	73.33
Cutoff > 4.00 [14, 31]	84.78	58.62	76.47	70.83	74.67
Cutoff > 6.50 [30]	71.74	86.21	89.19	65.79	77.33
Cutoff > 6.70 [6]	69.57	86.21	88.89	64.10	76.00
**Validation cohort (n = 37)**					
Cutoff > 5.30	76.19	81.25	84.21	72.22	78.38
Cutoff > 3.20 [11, 29]	100.00	18.75	61.76	100.00	64.86
Cutoff > 4.00 [14, 31]	80.95	50.00	68.00	66.67	67.57
Cutoff > 6.50 [30]	66.67	81.25	82.35	65.00	72.97
Cutoff > 6.70 [6]	66.67	80.00	82.35	63.16	72.22

Table S7. Change in GS between biopsy and RP surgery in patients with PCa.

Characteristic	Biopsy GS	RP GS	n (%)
No change (n = 39, 81.25%)	3 + 3 = 6	3 + 3 = 6	4/48 (8.33)
	3 + 4 = 7	3 + 4 = 7	5/48 (10.42)
	4 + 3 = 7	4 + 3 = 7	9/48 (18.75)
	4 + 4 = 8	4 + 4 = 8	9/48 (18.75)
	5 + 3 = 8	5 + 3 = 8	4/48 (8.33)
	4 + 5 = 9	4 + 5 = 9	3/48 (6.25)
	5 + 4 = 9	5+ 4 = 9	2/48 (4.17)
	5 + 5 = 10	5 + 5 = 10	3/48 (6.25)
Upgrade (n = 7, 14.58%)	3 + 3 = 6	3 + 4 = 7	1/48 (2.08)
	3 + 4 = 7	4 + 5 = 9	1/48 (2.08)
	4 + 3 = 7	4 + 4 = 8	1/48 (2.08)
	4 + 4 = 8	5 + 3 = 8	1/48 (2.08)
	4 + 4 = 8	4 + 5 = 9	2/48 (4.17)
	5 + 3 = 8	4 + 5 = 9	1/48 (2.08)
Downgrade (n = 2,4.17%)	4 + 4 = 8	4 + 3 = 7	1/48 (2.08)
	4 + 4 = 8	3 + 4 = 7	1/48 (2.08)

Table S8. Intraclass correlation coefficient of SUV_{max} measurement.

Intraclass correlation coefficient	95% confidence interval		Value	*P*
	Lower bound	Upper bound		
0.993	0.991	0.995	278.423	<0.001
